# Dally Proteoglycan Mediates the Autonomous and Nonautonomous Effects on Tissue Growth Caused by Activation of the PI3K and TOR Pathways

**DOI:** 10.1371/journal.pbio.1002239

**Published:** 2015-08-27

**Authors:** Ana Ferreira, Marco Milán

**Affiliations:** 1 Institute for Research in Biomedicine (IRB Barcelona), Barcelona, Spain; 2 Institucio Catalana de Recerca i Estudis Avançats (ICREA), Barcelona, Spain; University of Zurich, SWITZERLAND

## Abstract

How cells acquiring mutations in tumor suppressor genes outcompete neighboring wild-type cells is poorly understood. The phosphatidylinositol 3-kinase (PI3K)–phosphatase with tensin homology (PTEN) and tuberous sclerosis complex (TSC)-target of rapamycin (TOR) pathways are frequently activated in human cancer, and this activation is often causative of tumorigenesis. We utilized the Gal4-UAS system in *Drosophila* imaginal primordia, highly proliferative and growing tissues, to analyze the impact of restricted activation of these pathways on neighboring wild-type cell populations. Activation of these pathways leads to an autonomous induction of tissue overgrowth and to a remarkable nonautonomous reduction in growth and proliferation rates of adjacent cell populations. This nonautonomous response occurs independently of where these pathways are activated, is functional all throughout development, takes place across compartments, and is distinct from cell competition. The observed autonomous and nonautonomous effects on tissue growth rely on the up-regulation of the proteoglycan Dally, a major element involved in modulating the spreading, stability, and activity of the growth promoting Decapentaplegic (Dpp)/transforming growth factor β(TGF-β) signaling molecule. Our findings indicate that a reduction in the amount of available growth factors contributes to the outcompetition of wild-type cells by overgrowing cell populations. During normal development, the PI3K/PTEN and TSC/TOR pathways play a major role in sensing nutrient availability and modulating the final size of any developing organ. We present evidence that Dally also contributes to integrating nutrient sensing and organ scaling, the fitting of pattern to size.

## Abstract 2

The loss of tumor suppressor genes induces a nonautonomous reduction of growth and proliferation rates in adjacent cell populations by competing for available growth factors; the proteoglycan Dally helps to mediate this effect.

## Author Summary

The final size of a developing organ is finely modulated by nutrient conditions through the activity of nutrient sensing pathways, and deregulation of these pathways is often causative of tumorigenesis. Besides the well-known roles of these pathways in inducing tissue and cell growth, here we identify a nonautonomous effect of activation of these pathways on growth and proliferation rates and on the final size of neighboring cell populations. We reveal that the observed autonomous and nonautonomous effects on tissue growth and proliferation rates rely on the up-regulation of the proteoglycan Dally, a major factor involved in modulating the spreading, stability, and activity of the growth promoting Decapentaplegic (Dpp)/transforming growth factor β(TGF-β) signaling molecule. Our data indicate that a reduction in the amount of available growth factors contributes to the outcompetition of wild-type cells by overgrowing cell populations. Whereas nutrient-sensing pathways modulate the final size of the adult structure according to nutrient availability to the feeding animal, Dpp plays an organ-intrinsic role in the coordination of growth and patterning. We identify the proteoglycan Dally as the rate-limiting factor that contributes to the tissue-autonomous and nonautonomous effects on growth caused by targeted activation of the nutrient-sensing pathways. Thus, our results unravel a role of Dally as a molecular bridge between the organ-intrinsic and organ-extrinsic mechanisms that regulate organ size.

## Introduction

Several conserved signaling pathways involved in the control of organ size in many species are frequently deregulated in a broad range of human cancers, and this deregulation is often causative of tumorigenesis. The phosphatidylinositol 3-kinase (PI3K)–phosphatase with tensin homology (PTEN) is one of the most commonly altered pathways in human tumors. Deletion of PTEN is associated with aggressive metastatic potential and poor prognosis (reviewed in [1]), and targeted depletion of PTEN in epithelial cells leads to rapid development of endometrial, prostate, and thyroid neoplasias in mouse models [2]. Mutations in tuberous sclerosis complex 1 (TSC1) and tuberous sclerosis complex 2 (TSC2) that lead to hyperactivation of the target of rapamycin (TOR) pathway are causative of tuberous sclerosis, a hamartoma syndrome associated with a predisposition to malignancy [3], and heterozygous *Tsc1* or *Tsc2* mutant mice develop renal and extrarenal tumors such as hepatic hemangiomas [4].

In *Drosophila*, the PI3K/PTEN and TSC/TOR pathways modulate the final size of the developing organism according to nutrient availability, and their activation promotes cell and tissue growth (reviewed in [5,6]). Thus, the identification of the molecular players and cellular interactions underlying the induction of growth upon loss of these tumor suppressor genes in model organisms will bring new insight into their contribution to the initial steps of tumorigenesis, as somatic mutations in these pathways appear to be frequently accumulated in early events of tumor development. In this context, the imaginal primordia of *Drosophila*, monolayered epithelial tissues that grow and proliferate 1,000-fold in size in about five days inside the feeding larvae, have proved to be a valuable model system to address the impact of tumor suppressor genes in the early events of tumorigenesis (reviewed in [7,8]). Their simple architecture enables both the labelling and tracking of cell populations that can be genetically manipulated with the help of sophisticated tools as well as the analysis of the cell-autonomous and nonautonomous impact of loss of these tumor suppressor genes.

Of particular interest is the understanding of how cells depleted of tumor suppressor genes outcompete neighboring wild-type cells, especially during early stages of tumorigenesis following initial mutagenesis and transformation. Cell competition, a process favoring fit over weak or harmful cells, was originally identified in *Drosophila* [9] and has been proposed to play a protumoral role in this process [10]. However, cell competition has been recently shown to play a tumor suppressor role in the mammalian thymus as it prevents the selection of faulty cells, which become tumorigenic [11]. Thus, the initial proposal of cell competition as a driving force in tumor progression remains elusive. Work in *Drosophila* imaginal primordia has also shown that competition for nutrients contributes to the growth of *PTEN* mutant cells and the outcompetition of wild-type surrounding cells [12]. These results explain the resistance of tumors lacking PTEN or with increased PI3K activity to dietary restriction [13].

Here, we used the developing wing primordium of *Drosophila* to analyze the autonomous and nonautonomous impact of *PI3K/PTEN* and *TSC/TOR* pathway activation. We utilized the GAL4/ upstream activation sequence (UAS) system to target these pathways in restricted cell populations that correspond to the developing compartments—cell populations that do not mix and give rise to defined structures of the adult wing [14]. We present evidence that activation of these two pathways, well known to induce tissue growth in an autonomous manner, causes a nonautonomous reduction in the growth and proliferation rates as well as in the resulting tissue size of adjacent cell populations. The observed autonomous and nonautonomous effects on tissue growth rely on the up-regulation of the proteoglycan Dally, a major element involved in modulating the spread, stability, and activity of the growth-promoting Decapentaplegic (Dpp)/transforming growth factor β (TGF-β) signaling molecule. These results shed new light on the selection process that takes place during the early stages of tumorigenesis following initial mutagenesis and transformation, in which competition for secreted growth-promoting factors might play a fundamental role. During normal development, the PI3K/PTEN and TSC/TOR signaling pathways modulate the final size of the adult wing as a function of nutrient availability (reviewed in [15,16]), whereas Dpp plays an organ-intrinsic role in the coordination of growth and patterning within the developing wing primordium (reviewed in [17,18]). Our results also identify Dally as a molecular bridge between nutrient sensing and wing scaling, the latter defined as the fitting of pattern to size.

## Results

### Targeted Activation of the PI3K/PTEN or TSC/TOR Pathways Induces a Nonautonomous Reduction in Tissue Size of the Adjacent Cell Populations

**Fig 1:**
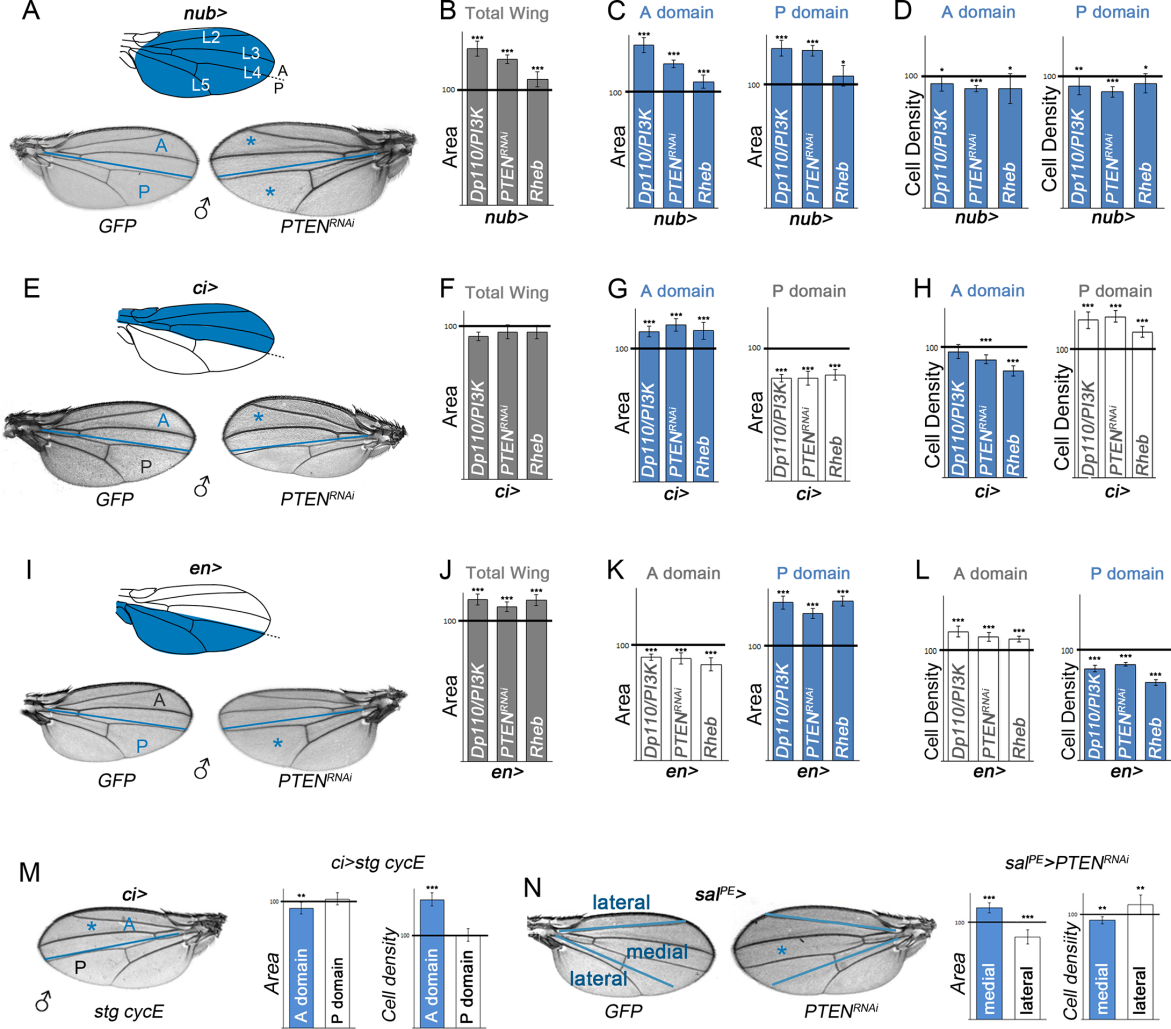
Nonautonomous effects on tissue size upon targeted activation of PI3K/PTEN and TSC/TOR pathways. (A, E, I) Schemes of adult wings with the *nub* (A), *ci* (E), and *en* (I) expression domains marked in blue and cuticle preparations of male adult wings expressing *GFP* or *PTEN* ^*RNAi*^ under the control of the *nub-gal4* (A), *ci-gal4* (E), and *en-gal4* (I) drivers. The blue line marks the boundary between anterior (A) and posterior (P) compartments. The wing is decorated with four longitudinal veins (L2–L5), and the anterior side of L4 corresponds to the AP compartment boundary. (B–D, F–H, J–L) Histograms plotting tissue size (B, C, F, G, J, K) and cell density (D, H, L) of the whole wing (B, F, J), or the A and P compartments of adult wings expressing the indicated transgenes in the *nub* (B–D), *ci* (F–H) or *en* (J–L) domains normalized as a percent of the control wings. Note a consistent reduction in tissue size of the adjacent cell populations in G and K (white bars). (M) Cuticle preparation of a *ci>stg*, *cycE* adult wing. The blue line marks the boundary between the A and P compartments. On the right are histograms plotting tissue size and cell density values of the A (blue bars) and P (white bars) domains of *ci>stg*, *cycE* adult wings normalized as a percent of the control (*ci>GFP*) wings. *ci-gal4* drives gene expression to the A compartment of the wing. (N) Cuticle preparations of *spalt* ^*PE*^ *>GFP* and *spalt* ^*PE*^ *>PTEN* ^*RNAi*^ adult wings. The blue lines mark the lateral and medial regions of the wing. On the right are histograms plotting tissue size and cell density values of the medial (blue bars) and lateral (white bars) regions of *spalt* ^*PE*^ *>PTEN* ^*RNAi*^ adult wings normalized as a percent of the control (*spalt* ^*PE*^ *>GFP*) wings. *spalt-gal4* drives gene expression to the medial part of the wing. The domains of transgene expression in A, E, I, M, and N are marked with a blue asterisk. Error bars show the standard deviation. Number of wings analyzed per genotype ≥ 10. ***p < 0.001; **p < 0.01; *p < 0.05. Area and cell density values of the cell populations driving transgene expression are labeled in blue, and the values of the nonexpressing domain are represented in white.

The activation of the PI3K/PTEN and TSC/TOR pathways induces tissue growth in the developing imaginal primordia and gives rise to overgrown adult structures [19–23]. Interestingly, these pathways exert this action by affecting cell size and cell proliferation in different manners. Activation of the PI3K/PTEN pathway in the whole wing (with the *nubbin-gal4* driver, Fig 1A), either by expression of *PI3K-92E* (also termed *Drosophila p110* or *Dp110*) or a double-stranded RNA (dsRNA) form of *PTEN* (a negative regulator of PI3K/Dp110), gave rise to larger wings than those of control flies expressing green fluorescent protein (GFP) (Fig 1A and 1B). The increase in tissue size was a consequence of both an increase in cell size (reflected by a reduction in cell densities in the adult wing, Fig 1D) and in cell number (note the increase in tissue size in Fig 1B and 1C is much larger than the reduction in cell densities in Fig 1D). Activation of the TOR pathway, by depletion of *TSC1 or TSC2*, leads to strongly overgrown tissues and larval lethality [24]. In order to reduce larval lethality and to analyze the resulting adult wings, we induced mild activation of the TOR pathway by overexpressing the guanosine-5'-triphosphate (GTP)-binding protein Rheb, which binds and activates TOR [25–27]. Rheb overexpression gives rise to overgrown adult wings (Fig 1B). The observed increase in tissue size is mainly a consequence of an increase in cell size (Fig 1D, note the reduction in cell densities is to a similar extent as the increase in tissue size shown in Fig 1B and 1C). The adult wing is subdivided into an anterior (A) and a posterior (P) compartment, and the boundary between these two cell populations is easily identified in the adult wing (Fig 1A), as it corresponds to the anterior side of the fourth longitudinal vein (L4, Fig 1A). The observed increase in tissue size upon activation of the PI3K/PTEN or TSC/TOR pathways in the whole wing was similar in both compartments (Fig 1C).

To address the potential nonautonomous roles of the *PTEN* and *TSC* tumor suppressor genes on neighboring wild-type cell populations, we drove transgene expression in either the A (with the *ci-gal4* driver) or P (with the *en-gal4* or *hh-gal4* drivers) compartments of the developing *Drosophila* wing (Fig 1E and 1I and S1 Fig) and quantified the size of the adjacent compartments in the resulting adult structures. The autonomous capacity of these transgenes to induce tissue and/or cell growth and cell proliferation was also observed with these Gal4 drivers (Fig 1E, 1G, 1H and 1K and 1L and S1 Fig, blue bars). Interestingly, the size of neighboring wild-type tissues decreased in all cases (Fig 1E, 1G, 1I and 1K and S1 Fig, white bars), and this decrease was accompanied by a reduction in cell size (reflected by an increase in cell densities in the adult wing (Fig 1H and 1L and S1 Fig, white bars). Whether the reductions in cell and tissue size are two independent processes nonautonomously regulated by the PI3K/PTEN and TSC/TOR pathways or whether there is a causal relationship between them is further analyzed in the following sections. The nonautonomous effects on tissue growth were also obtained with other Gal4 drivers not specific to compartments (Fig 1N). Increased cell proliferation, by means of overexpression of the cell cycle regulators String/Dcdc25 and CycE (which drive G2/M and G1/S transitions, respectively [28–30]), gave rise to a higher number of cells as a consequence of increased mitotic activity (Fig 1M and S1 Fig, see also [31]) but was not sufficient to promote tissue growth (Fig 1M, blue bar). The transgene-expressing compartment was, if anything, slightly smaller (Fig 1M, blue bar), most probably because of the observed increase in the number of apoptotic cells in the tissue (S1 Fig, see also [31]). Thus, the capacity of PI3K/PTEN to promote tissue growth does not depend only on increased cell proliferation rates. Driving cell proliferation was not sufficient to exert any nonautonomous effects on tissue or cell growth either (Fig 1M, white bars). All together, these results indicate that tissue overgrowth, induced by different means and independently of whether it is a consequence of increased cell size and/or cell number, causes a nonautonomous reduction in tissue size of adjacent cell populations.

### Targeted Activation of the PI3K/PTEN or TSC/TOR Pathways Induces a Nonautonomous Reduction of Growth Rates in Adjacent Cell Populations

**Fig 2:**
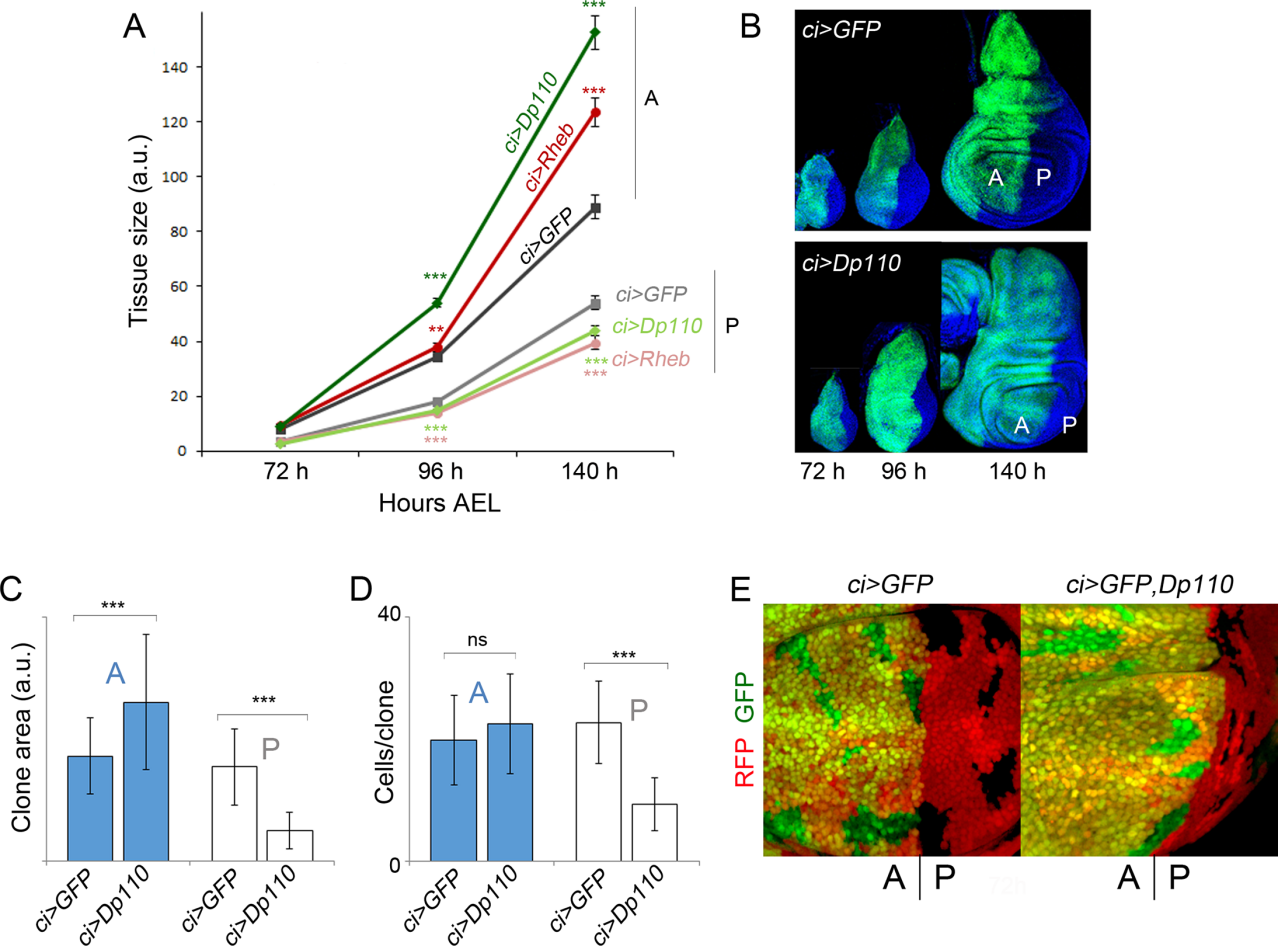
Targeted activation of the PI3K/PTEN and TSC/TOR pathways induces a nonautonomous reduction in growth rates in adjacent cell populations. (A) Quantification of tissue size (in arbitrary units [a.u.]) of both anterior (A) and posterior (P) domains of wing imaginal discs of the indicated genotypes in three distinct time points during development (in hours [h] AEL). Error bars show the standard deviation. Number of wing discs analyzed per genotype ≥ 15. ***p < 0.001; **p < 0.01. (B) Examples of *ci>GFP* (upper panel) and *ci>GFP*, *Dp110* (lower panel) wing imaginal discs in the developmental time points shown in panel A. The transgene-expressing domain is marked with GFP (in green), and the disc is labelled with DAPI (in blue). The A and P compartments are indicated. (C, D) Histograms plotting the size of clones (in a.u., C) and the number of cells per clone (D) located in the A or P compartment of *ci-gal4*,*UAS-GFP* and *ci-gal4*, *UAS-GFP/UAS-Dp110* wing discs. Clones were generated at the beginning of the third instar period and quantified 72 h later in late third instar wing discs. Error bars indicate the standard deviation. Number of clones analyzed per genotype ≥ 30. ***p < 0.001. Size of clones (A compartment, a.u.): ci>GFP = 388 ± 141; ci>GFP, Dp110 = 587 ± 249. Size of clones (P compartment, a.u.): ci>GFP = 348 ± 140; ci>GFP, Dp110 = 114 ± 67. Number of cells per clone (A compartment): ci>GFP = 20 ± 7; ci>GFP, Dp110 = 23 ± 8. Number of cells per clone (P compartment): ci>GFP = 23 ± 7; ci>GFP, Dp110 = 9 ± 4. (E) Examples of clones of cells in *ci>GFP* (left panel) and *ci>GFP*, *Dp110* (right panel) wing discs and induced in the developmental time points shown in C and D. The transgene-expressing domain is marked with GFP (in green), and the clones are labeled by the absence of nuclear red fluorescent protein (RFP, red) expression. The A and P compartments are indicated.

The results presented so far indicate that induction of tissue overgrowth in defined territories of the wing primordia causes a nonautonomous decrease in tissue size of nearby cell populations. To understand whether this nonautonomous response is an active mechanism that takes place during development, we monitored the size of the A and P compartments throughout development after targeted expression of growth-promoting transgenes in anterior cells. Larvae expressing PI3K/Dp110, Rheb, or GFP in the A compartment (with the *ci-gal4* driver) were grown at 25°C, and the absolute size of the transgene-expressing (A) and nonexpressing (P) compartments was quantified at a range of time points of larval development. We focused our attention on third instar wing discs (from 72 to 140 h after egg laying [AEL]), as primordia of the future adult wings are specified in late second instar (around 60 h AEL, [32]). The autonomous impact of PI3K/Dp110 or Rheb on tissue growth was already visible in mid–late third instar wing discs (96–140 h AEL), and, most interestingly, this was accompanied by a clear reduction in tissue size of the neighboring P compartment (Fig 2A and 2B). Thus, activation of growth promoting pathways in one territory causes a nonautonomous reduction in growth rates of the adjacent cell population.

In order to investigate whether cell proliferation rates are also regulated in a nonautonomous manner, we induced neutral clones of cells at the beginning of the third instar period (72 h AEL) and examined the size of these clones 72 h later in late third instar wing discs. Clone size (in arbitrary units [a.u.]) and number of cells per clone were measured in *ci-gal4; UAS-GFP/UAS-Dp110* wing discs, and these two measurements were compared to those of control clones induced in *ci-gal4; UAS-GFP/+* wing discs and grown in parallel. In *Dp110*-expressing wing discs, the size of the clones in the A compartment was, as expected, significantly larger than the size of clones quantified in the A compartment of GFP-expressing discs (Fig 2C–2E). Interestingly, the clone size and the number of cells per clone were significantly smaller in the P compartment of *Dp110*-expressing wing discs than in GFP-expressing discs (Fig 2C–2E). We also used a fluorescence-activated cell sorter (FACS) to collect data about the DNA content of dissociated cells from 120 h AEL wing discs expressing Dp110 or GFP under the control of the *ci-gal4* driver. As shown in S1 Fig, the cell cycle profile of A and P cells was very similar in the two genotypes analyzed. Taken together, these results indicate that activation of growth promoting pathways causes a nonautonomous reduction in proliferation rates, without any obvious arrest in any particular cell cycle stage, and imply that the nonautonomous reduction in tissue size observed in adult wings is a consequence of not only reduced cell size but also cell number.

### Nonautonomous Effects on Tissue Growth Are Independent of Apoptosis and Dietary Restriction

**Fig 3:**
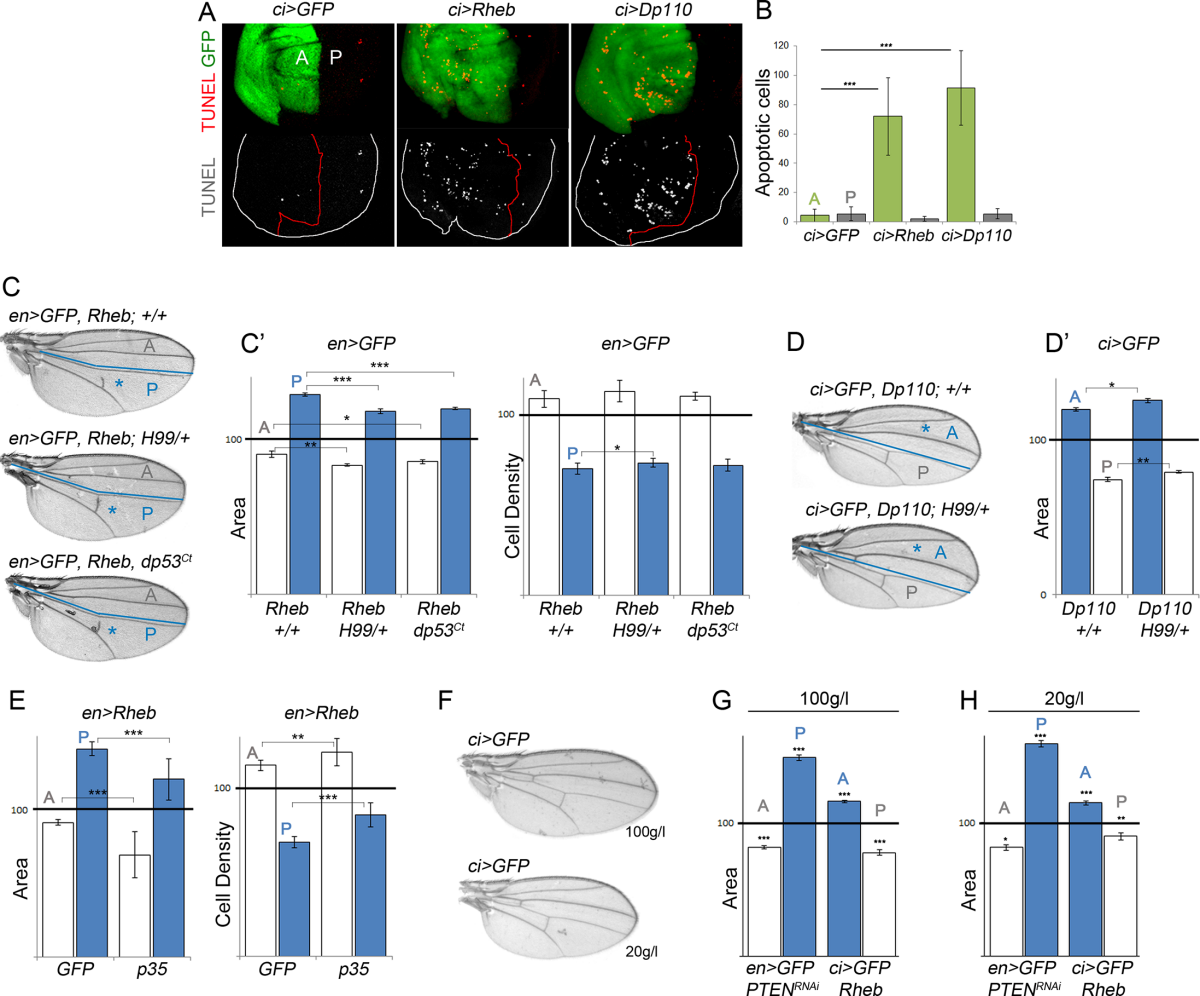
The nonautonomous reduction in tissue size upon targeted activation of the PI3K/PTEN and TSC/TOR pathways does not rely on Dp53 activity and apoptosis and is not affected by nutrient restriction. (A) *ci>GFP*, *ci>Rheb*, and *ci>Dp110* wing imaginal discs labelled with TUNEL to visualize apoptotic cells (in red or white). The *ci* domain is labelled with GFP (in green), and the boundary between A and P cells is marked by a red line. (B) Histogram plotting the quantification of the absolute number of TUNEL-positive cells in the A (light green bars) and P (grey bars) compartments of the indicated genotypes. Error bars indicate the standard deviation. Number of wing discs analyzed per genotype ≥ 10. ***p < 0.001. (C, D) Cuticle preparations of *en>GFP*, *Rheb* (C), and *ci>GFP*, *Dp110* (D) adult wings either in a wild-type background, in a heterozygous background for *H99* (a deficiency that uncovers *reaper*, *hid*, and *grim* proapoptotic genes), or coexpressing a dominant negative form of Dp53 (Dp53^Ct^). The blue line marks the boundary between the A and P compartments. The domains of transgene expression are marked with a blue asterisk. (C’, D’, E) Histograms plotting tissue size (C’, D’, E) and cell density values (C’, E) of the transgene-expressing compartment (blue bars) and the adjacent cell populations (white bars) of adult wings with the indicated genotypes normalized as a percent of the control wings. Error bars show the standard deviation. Number of wings analyzed per genotype ≥ 10. ***p < 0.001; **p < 0.01; *p < 0.05. (F) Cuticle preparations of *ci>GFP* adult wings of well-fed (100 g/L yeast food, top) and starved (20 g/L yeast food, bottom) animals. Note the reduction in wing size caused by starvation. (G, H) Histograms plotting tissue size of the transgene-expressing compartment (blue bars) and the adjacent cell populations (white bars) of adult wings with the indicated genotypes normalized as a percent of the control wings. Error bars show the standard deviation. Number of wings analyzed per genotype ≥ 10. ***p < 0.001; **p < 0.01; *p < 0.05. Quantification was made in well-fed (100 g/L yeast food, left histogram) and starved (20 g/L yeast food, right histogram) animals of identical genotypes.

The nonautonomous reduction in growth rates caused by activation of the PI3K/PTEN or TSC/TOR pathways in defined cell populations of the developing wing primordia is reminiscent of cell competition, a short-range elimination of slow-dividing cells through apoptosis when confronted with a faster-growing cell population [33]. We thus performed a terminal deoxynucleotidyl transferase dUTP nick end labeling (TUNEL) assay to label DNA strand breaks induced by apoptotic cell death and quantified the number of apoptotic cells in the transgene-expressing and nonexpressing cell populations. A clear increase in TUNEL-positive cells was observed in those territories expressing Rheb or Dp110/PI3K when compared to GFP-expressing tissues (Fig 3A and 3B, green bars). A similar cell autonomous induction of apoptosis was previously observed in *PTEN* mutant cells [12], most probably reflecting a deleterious effect of nonphysiological activation of these pathways in proliferating cells. However, the number of apoptotic cells in adjacent cell populations was small and very similar to that found in control tissues expressing GFP (Fig 3A and 3B, grey bars, see also [34]). We next analyzed a potential contribution of apoptotic cell death to the nonautonomous reduction in growth rates caused by Rheb or Dp110/PI3K expression. When apoptosis was reduced in the whole animal by halving the dose of the proapoptotic genes *hid*, *grim*, and *reaper* (in *Df(H99)/+* larvae, [35]), the number of apoptotic cells in the tissue was reduced (S1 Fig), but the nonautonomous reduction in tissue size was unaffected (Fig 3C, 3C’, 3D and 3D’). The transcription factor and tumor suppressor p53, a short-lived, nonabundant protein in healthy cells, plays a fundamental role in regulating the response of mammalian cells to stress, in part through the transcriptional activation of genes involved in apoptosis and cell cycle regulation [36]. Impaired protein translation in *Drosophila* tissues has been previously shown to induce Dp53 activation and Dp53-dependent inhibition of growth and proliferation rates in adjacent tissues [37]. However, the nonautonomous effects on tissue growth caused by Rheb or Dp110/PI3K overexpression were unaffected by expression of a dominant negative version of Dp53 (Dp53^ct^, Fig 3C and 3C’). Taken together, all these results indicate that Dp53 is not required in the overgrown territory to nonautonomously reduce the growth rates in neighboring cell populations and that the nonautonomous decrease in growth rates is not a consequence of programmed cell death.

Apoptotic cells can produce signals to instruct neighboring cells either to undergo additional proliferation or apoptosis [34,38–40]. We thus analyzed whether the apoptotic cells observed in the transgene-expressing compartment exerted any nonautonomous effect on the neighboring compartment. Expression of p35, a baculovirus protein well known to block apoptosis at the level of the effector apoptotic Caspases [41], was not able to rescue the nonautonomous decrease in tissue growth caused by Rheb overexpression (Fig 3E). We found out that p35 expression caused an increase in cell density in both the transgene-expressing compartment and in the neighboring one, most probably reflecting apoptosis-induced cell proliferation caused by undead cells. Indeed, mitotic activity was increased in the developing primordia expressing p35 (S1 Fig). These results indicate that dying cells in the transgene-expressing compartment do not contribute to the nonautonomous reduction in tissue and cell size.

*PTEN* mutant cells generated in *Drosophila* tissues have been recently shown to acquire a growth advantage under starvation conditions at the expense of the wild-type neighboring cells, and this growth advantage has been proposed to rely on the withdrawal of nutrients from neighboring cells [12]. To assess the impact of nutrient availability on the autonomous and nonautonomous effects caused by expression of Rheb or *PTEN-RNAi* in defined territories of the wing primordium, larvae expressing these transgenes in either the A or P compartment were reared on food with varying yeast content, the main source of micronutrients and amino acids in fly media. Our standard medium contains 50 g/l yeast. We thus subjected experimental larvae expressing either a dsRNA form of PTEN or Rheb and control larvae expressing GFP to media containing 100 g/l and 20 g/l yeast and analyzed the size of the transgene-expressing and nonexpressing cell populations in the resulting adult wings. Despite the expected reduction in the size of wings of animals reared on food containing 20 g/l yeast (Fig 3F), the extent (in percentage) of the nonautonomous decrease in tissue size caused by transgene expression was similar to that observed in animals reared on food containing 100 g/l yeast (Fig 3G and 3H). Thus, the nonautonomous reduction in growth rates is not a consequence of the withdrawal of nutrients from neighboring cells.

### Autonomous and Nonautonomous Effects on the Dpp Activity Gradient

**Fig 4:**
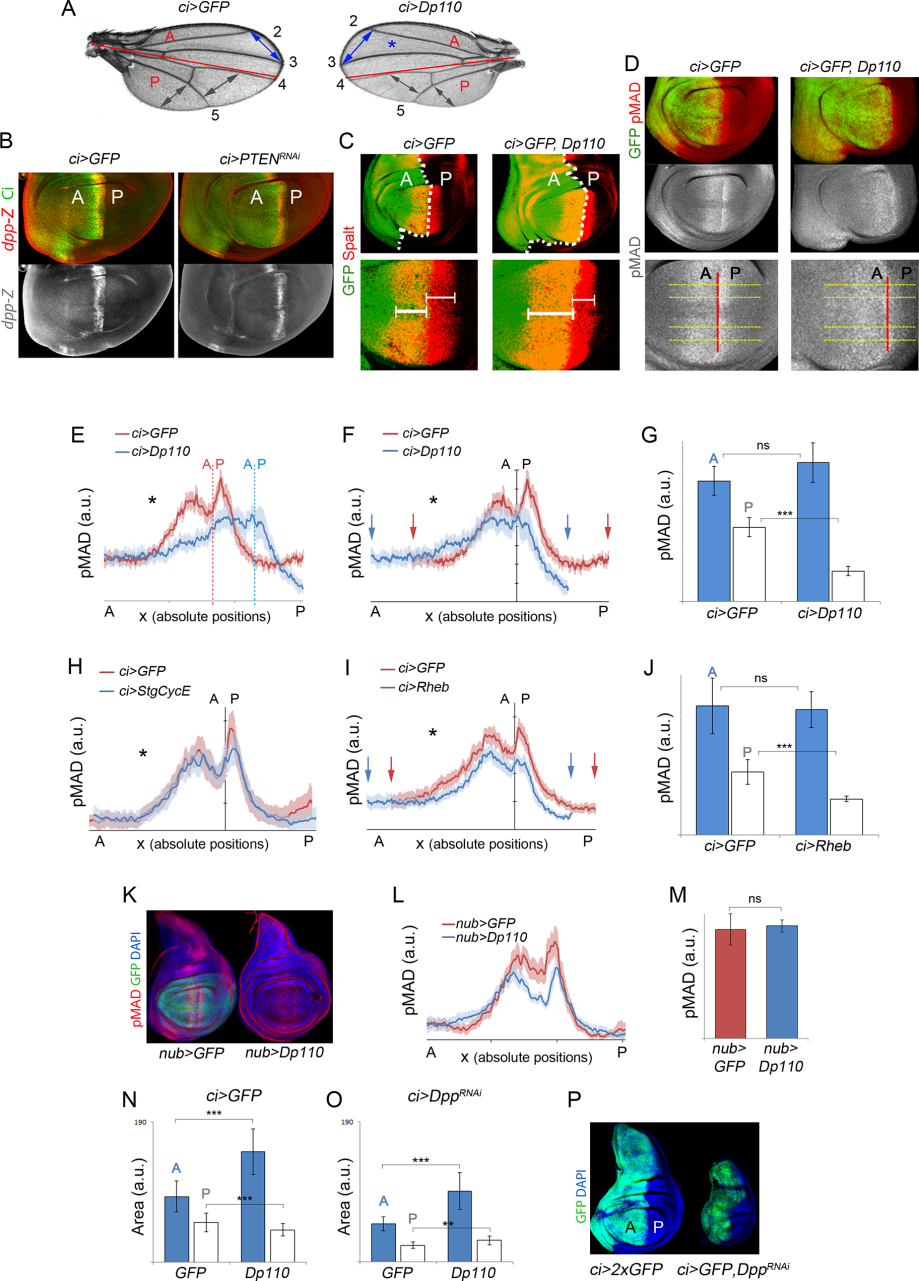
Autonomous and nonautonomous effects on Dpp activity levels upon targeted activation of the PI3K/PTEN and TSC/TOR pathways. (A) Cuticle preparation of adult wings expressing *GFP* or *Dp110* under the control of the *ci-gal4* driver, which drives transgene expression in the anterior compartment. Note the expansion of the anterior intervein regions (blue arrows) upon induction of growth and the corresponding reduction of the posterior intervein regions (grey arrows). The anterior (A) and posterior (P) compartments are labeled, and the AP boundary is marked by a red line. (B) Wing imaginal disc of *ci>GFP* and *ci>PTEN* ^*RNAi*^ larvae labelled to visualized Ci protein (in green) and β-galactosidase (βGal) (in red or white) to visualize *dpp* (dpp-lacZ) expression. The width of the *dpp-lacZ* stripe (normalized with respect to the width of the wing pouch) was *ci>GFP* = 0.082 ± 0.005; *ci>PTEN* ^*RNAi*^ = 0.088 ± 0.003; *p* = 0.55; *n* > 6. (C) Wing imaginal discs of *ci>GFP* and *ci>GFP*, *Dp110* larva labelled to visualize Spalt (in red) and GFP (in green) protein expression. The white dashed line marks the boundary between A and P cells. The lower panels show magnification of the Spalt domain. Note that the anterior Spalt domain is wider upon Dp110 expression (thick bracket) whereas the posterior Spalt domain (thin brackets) gets reduced. (D) Wing imaginal discs of *ci>GFP* and *ci>GFP*, *Dp110* larva labelled to visualize pMAD protein (in red or white) and GFP (in green). Lower panels show magnifications of the pMAD domains, and the red line marks the boundary between the A and P compartments. Horizontal yellow lines were used to generate the pMAD profiles shown in E–I. (E, F, H, I) Average pMAD profiles of wing discs expressing *GFP* (red line) or *GFP* and the corresponding transgenes (blue line) under the control of the *ci-gal4* driver. Profiles were taken along the AP axis and plotted in absolute positions. The standard error to the mean is shown in the corresponding color for each genotype. In E, the AP boundary of each experiment is marked by a dashed line of the corresponding color. In F, the AP boundary of both experiments was aligned to allow comparison of the profile in each compartment. Number of wing discs analyzed per genotype ≥ 5. The domains of transgene expression are marked with a black asterisk. Arrows mark the limits of the Dpp activity gradients. (G, J) Histograms plotting the total intensity of the pMAD signal in a.u. of the anterior (blue bars) and posterior (white bars) compartments of *ci>GFP* (G, J) and *ci>Dp110* (G) or *ci>Rheb* (J) wing discs. Error bars indicate the standard deviation. Number of wing discs analyzed per genotype ≥ 5. ***p < 0.001. (K) Wing imaginal discs of *nub>GFP* and *nub>Dp110* larvae labelled to visualize pMAD protein (in red), GFP (in green), and DAPI (in blue, to visualize nuclei). (L) Average pMAD profile of wing discs expressing *GFP* (red line) or *Dp110* (blue line) in the *nubbin* domain. Profiles were taken along the AP axis and plotted in absolute positions. The standard error to the mean is shown in the corresponding color for each genotype. Number of wing discs analyzed per genotype ≥ 7. (M) Histogram plotting the total intensity of the pMAD signal in a.u. of the *nubbin* domain of *nub>GFP* (red bar) and *nub>Dp110* (blue bar) discs. Error bars indicate the standard deviation. Number of wing discs analyzed per genotype ≥ 5. (N) Histogram plotting the area (in a.u.) of anterior (blue) and posterior (white) domains of wing discs expressing *GFP* or *GFP* and *Dp110* in the *ci* domain. Error bars show the standard deviation. Number of wing discs analyzed per genotype ≥ 15. ***p < 0.001 (O) Histogram plotting the area (in a.u.) of anterior (blue) and posterior (white) domains of wing discs expressing either *GFP* or *Dp110* in the *ci* domain together with a dsRNA form against *dpp*. Error bars show the standard deviation. Number of wing discs analyzed per genotype ≥ 15. ***p < 0.001, **p < 0.01. (P) Wing imaginal discs of *ci>GFP* and *ci>dpp* ^*RNAi*^ larvae labelled to visualize GFP (in green) and DAPI (in blue, to visualize nuclei).

We noticed that activation of the PI3K/PTEN or TSC/TOR pathways in defined populations of the wing primordium gave rise to larger but well-proportioned adult structures (Fig 4A, see also Fig 1). The adjacent wild-type territories were reduced in size, but the patterning elements (e.g., longitudinal veins) were also proportionally well located. The signaling molecule Dpp, a member of the TGF-β superfamily, is expressed at the boundary between the A and P compartments in the developing wing (Fig 4B) and plays a major role in positioning the wing veins along the anterior–posterior axis [42]. Interestingly, the Dpp gradient scales with size in order to correctly maintain the wing proportions [43–46], and Dpp is well known to promote tissue growth (reviewed in [17,18]). Thus, the autonomous and nonautonomous effects of Rheb or Dp110/PI3K overexpression on tissue growth might rely on modulating the range of Dpp activity. Since *dpp* expression was largely unaffected in transgene-expressing tissues (Fig 4B), we monitored the range of the Dpp activity gradient, visualized by the expression of the Dpp target gene Spalt (Fig 4C, [47]) and with an antibody against the phosphorylated form of the Dpp transducer Mothers Against Dpp (pMAD, Fig 4D). As expected and as a result of the capacity of the Dpp gradient to scale with tissue size, the range of the Dpp activity gradient was expanded in the overgrowing transgene-expressing compartment (Fig 4C and 4D). In order to quantify the cell-autonomous impact of tissue growth on the Dpp activity gradient, we extracted the pMAD profiles along four lines perpendicular to the *dpp* expression domain (yellow lines in Fig 4D) and plotted the average pMAD values along the AP axis in experimental (blue in Fig 4E, 4F, 4H and 4I) and control (red in Fig 4E, 4F, 4H and 4I) wing primordia raised in the same conditions and immunolabeled in the same tube (as previously done in [45]). Since the boundary between A and P cells is out of phase in control and Dp110-expressing wing discs (Fig 4E), the pMAD profiles were aligned with respect to the AP boundary to better visualize the autonomous and nonautonomous effects on the Dpp activity gradient (Fig 4F and 4I). Despite the visible expansion along the AP axis of the pMAD gradient in the overgrowing compartment (black asterisk in Fig 4E, 4F and 4I, see also S2 Fig), the total amount of pMAD, quantified as total pixel intensity within each compartment (see Materials and Methods), was comparable in overgrowing and control compartments (Fig 4G and 4J, see also S2 Fig). A similar relationship between the range of the pMAD gradient and the total amount of pMAD was observed when transgene expression was driven in the whole wing primordium (Fig 4K–4M). These results indicate that tissue growth has a major impact in Dpp spreading but not signaling. Two observations suggest that the tissue-autonomous expansion of the pMAD gradient is a consequence of increased tissue size, and that this expansion is not solely a consequence of increased number of cells. First, Rheb overexpression increased tissue and cell size, but not cell number, and led to the expansion of the pMAD gradient (Fig 4I, black asterisk). Second, increased cell number without affecting tissue size (by means of expression of the cell cycle regulators CycE and String/Dcdc25) did not have any impact on the pMAD gradient (Fig 4H, black asterisk).

Interestingly, both the width of the Dpp activity gradient, monitored by the expression of Spalt (Fig 4C) and pMAD (Fig 4D, 4E, 4F and 4I and S2 Fig), and the total amount of pMAD signaling (Fig 4G and 4J and S2 Fig) were reduced in adjacent wild-type territories. This nonautonomous effect was not observed upon expression of cell cycle regulators CycE and String/Dcdc25 (Fig 4H). We next addressed whether the nonautonomous reduction in tissue size relied on the capacity of the tissue to compete for the Dpp ligand. For this purpose, we compared the size of PI3K/Dp110-expressing and nonexpressing compartments upon depletion of Dpp, by means of expression of a dsRNA form against *dpp* in the A compartment (Fig 4N and 4O). Expression of a dsRNA form against *dpp* in A cells gave rise to a strong reduction in tissue size (Fig 4P). Whereas PI3K/Dp110 was still able to induce overgrowth of the transgene-expressing territory (Fig 4N and 4O, blue bars), the nonautonomous reduction in tissue size was largely rescued (compare Fig 4N and 4O, white bars). Taken together, these results indicate that tissue growth has a major impact in Dpp spreading but not signaling and that the nonautonomous reduction in tissue size is most probably a consequence of reduced Dpp signaling.

### Dally Contributes to the Autonomous and Nonautonomous Effects on Tissue Growth

**Fig 5:**
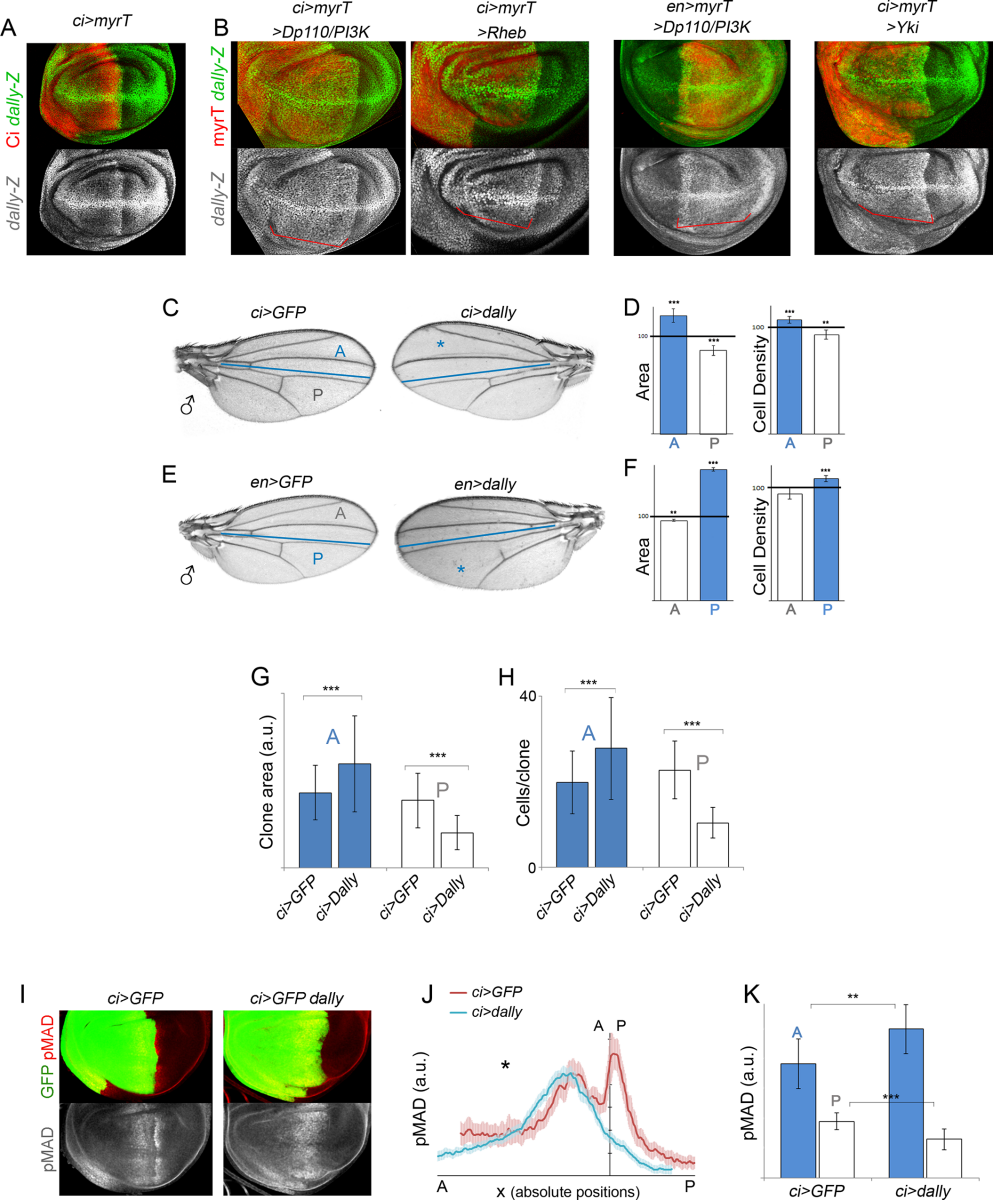
Overexpression of Dally phenocopies the autonomous and nonautonomous effects of targeted activation of the PI3K/PTEN and TSC/TOR pathways. (A, B) Representative wing discs of the indicated genotypes labeled to visualize *dally-lacZ* (antibody to β-Gal, green or white) and myrTomato (myrT, in red) expression to mark the transgene-expressing domain. Note stronger expression of *dally-lacZ* upon activation of the PI3K/PTEN, TSC/TOR, or hippo/Yorkie pathways (red brackets). (C, E) Cuticle preparations of male adult wings overexpressing Dally under the control of the *ci-gal4* (C) or *en-gal4* (E) drivers. The blue line marks the boundary between the anterior (A) and posterior (P) compartments, and the domains of Dally overexpression are marked with a blue asterisk. (D, F). Histograms plotting tissue size (left) and cell density values (right) of the Dally-expressing domains (blue bars) and the adjacent compartments (white bars) of adult wings overexpressing Dally under the control of the *ci-gal4* (D) or *en-gal4* (F) drivers. Values are normalized as a percent of the control (GFP-expressing) wings. Error bars show the standard deviation. Number of wings analyzed per genotype ≥ 10. ***p < 0.001; **p < 0.01. (G, H) Histograms plotting the size of clones (in a.u., G) and the number of cells per clone (H) located in the A or P compartment of *ci-gal4*, *UAS-GFP* and *ci-gal4*, *UAS-GFP/UAS-Dally* wing discs. Clones were generated at the beginning of the third instar period and quantified 72 h later in late third instar wing discs. Error bars indicate the standard deviation. Number of clones analyzed per genotype ≥ 30. ***p < 0.001. Size of clones (A compartment, a.u.): ci>GFP = 388 ± 141; ci>GFP, Dally = 537 ± 169. Size of clones (P compartment, a.u.): ci>GFP = 348 ± 140; ci>GFP, Dally = 182 ± 89. Number of cells per clone (A compartment): ci>GFP = 20 ± 7; ci>GFP, Dally = 28 ± 12. Number of cells per clone (P compartment): ci>GFP = 23 ± 7; ci>GFP, Dally = 10 ± 4. (I) Wing imaginal discs of *ci>GFP* and *ci>GFP*, *Dally* larvae labelled to visualize pMAD protein (in red or white) and GFP (in green). (J) Average pMAD profiles of wing discs expressing *GFP* (red line) or *GFP* and *Dally* (blue line) under the control of the *ci-gal4* driver. Profiles were taken along the AP axis and plotted in absolute positions. The standard error to the mean is shown in the corresponding color for each genotype. The AP boundary of both experiments was aligned to allow comparison of the profile in each compartment. Number of wing discs analyzed per genotype ≥ 5. (K) Histogram plotting the total intensity of the pMAD signal in a.u. of the anterior (blue bars) and posterior (white bars) compartments of *ci>GFP* and *ci>Dally*, *GFP* wing discs. Error bars indicate the standard deviation. Number of wing discs analyzed per genotype ≥ 5. ***p < 0.001; **p < 0.01.

Heparan sulfate proteoglycans (HSPGs) of the glypican family play key roles in the regulation of morphogen signaling and distribution (reviewed in [48]). Glypicans are glycosylphosphatidylinositol (GPI)-anchored HSPGs that have a protein core to which heparan sulfate (HS) chains are covalently attached. HS chains provide binding sites for many growth factors, and the glypican Dally contributes to the spreading of the Dpp ligand over the wing primordium [49–52]. Interestingly, the expression levels of Dally (visualized with two distinct reporters, an enhancer-trap and a proteintrap, see Fig 5A and 5B and S2 Fig) were clearly increased upon activation of the PI3K/PTEN or TSC/TOR pathways. We next monitored the capacity of targeted overexpression of Dally to phenocopy the autonomous and nonautonomous effects on tissue growth, proliferation rates, and Dpp gradient formation observed upon activation of these two pathways. Overexpression of Dally in the A or P compartments of the developing wing gave rise to a tissue-autonomous increase in size (Fig 5C–5F, blue bars) and, most importantly, a nonautonomous reduction in size of the adjacent wild-type territories (Fig 5C–5F, white bars). The increase in tissue size was accompanied by an increase in cell number (note the higher cell density in the transgene expressing compartment, Fig 5D, blue bars). In contrast to the PI3K/PTEN and TSC/TOR pathways, the nonautonomous reduction in tissue size caused by Dally overexpression was not accompanied by a decrease in cell size (note that cell densities, if anything, decreased, Fig 5D and 5F, white bars). Thus, other elements regulated by the growth-promoting pathways, apart from Dally, might have a nonautonomous impact on cell size. In order to address whether growth and proliferation rates are also regulated in a nonautonomous manner by the overexpression of Dally, we induced neutral clones of cells at the beginning of the third instar period (72 h AEL) and examined the size of these clones 72 h later in late third instar wing discs. Clone size and number of cells per clone were measured in the A and P compartments of *ci-gal4; UAS-GFP/UAS-Dally* wing discs, and these two measurements were compared to those of control clones induced in *ci-gal4; UAS-GFP/+* wing discs and grown in parallel. In Dally-overexpressing wing discs, the size of the clones and the number of cells per clone in the A compartment were significantly bigger than the size of clones quantified in the A compartment of GFP-expressing discs (Fig 5G and 5H). Interestingly, clone size and number of cells per clone were significantly smaller in the P compartment of Dally-expressing wing discs than in GFP-expressing discs (Fig 5G and 5H). These results indicate that overexpression of Dally causes a nonautonomous reduction in proliferation rates and imply that the nonautonomous reduction in tissue size observed in adult wings is a consequence of reduced cell number. Targeted overexpression of Dally also gave rise to a tissue-autonomous increase in the width of the pMAD signaling gradient and to a remarkable reduction in pMAD signaling in nearby territories (Fig 5I–5K). In this case, the total amount of pMAD was higher in Dally-overexpressing compartments than in the control one (Fig 5K). We noticed that the patterning elements, including wing veins and the wing margin, were well located and formed, thus indicating that Dally overexpression exerts a specific role on Dpp gradient formation and a minor role on the other secreted signaling molecules involved in wing growth and patterning.

**Fig 6:**
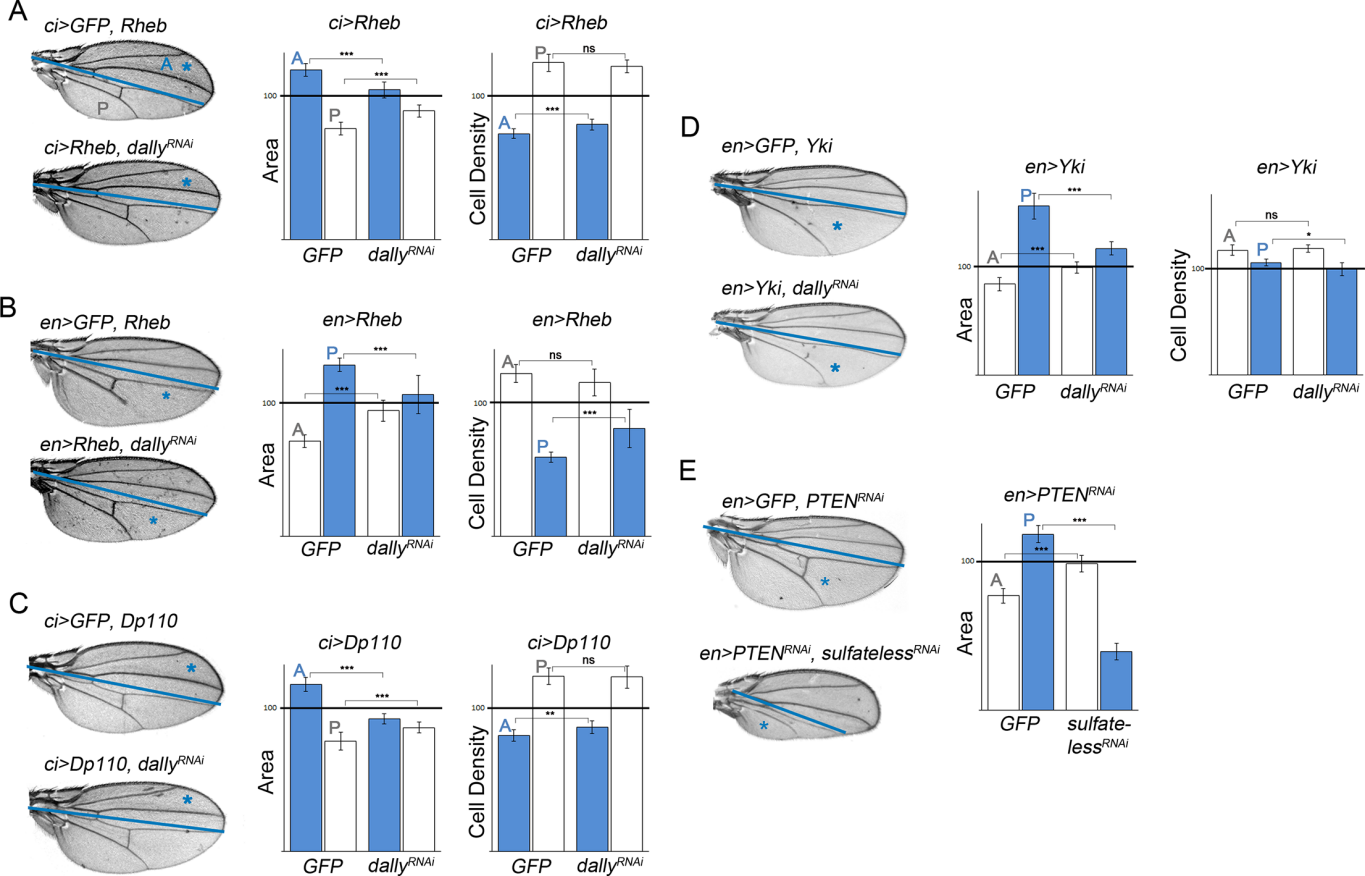
Dally contributes to the autonomous and nonautonomous effects of targeted deregulation of the PI3K/PTEN, TSC/TOR, and hippo/yorkie pathways. (A–E) Cuticle preparations of adult wings expressing the indicated transgenes under the control of the *ci-gal4* (A, C) or *en-gal4* (B, D, E) drivers. The blue line marks the boundary between the anterior (A) and posterior (P) compartments, and the domains of transgene expression are marked with a blue asterisk. On the right side of each panel, histograms plotting tissue size (A–E) and cell density (A–D) of the transgene-expressing (blue bars) or nonexpressing (white bars) compartments of adult wings expressing the indicated transgenes. Error bars show the standard deviation. Number of wings analyzed per genotype ≥ 10. ***p < 0.001; ** *p* < 0.01; *p < 0.05.

We next monitored the contribution of Dally to the autonomous and nonautonomous effects on tissue growth caused by targeted deregulation of the PI3K/PTEN and TSC/TOR pathways. For this purpose, we expressed a dsRNA form of *Dally* together with Dp110/PI3K or Rheb. Expression of a dsRNA form of *Dally* in otherwise wild-type wings caused a mild tissue-autonomous size reduction (S2 Fig, blue bars), but no visible effect was observed in adjacent cell territories (S2 Fig, white bars). The width of the Dpp activity gradient was mildly reduced in the transgene-expressing compartment (S2 Fig). Interestingly, depletion of Dally was able to largely rescue not only the autonomous but also the nonautonomous effects on tissue growth caused by Rheb or PI3K/Dp110 expression (Fig 6A–6C). The autonomous expansion of the Dpp activity gradient and the nonautonomous retraction caused by targeted expression of Dp110 was also largely rescued by the dsRNA form of *Dally* (S2 Fig). Depletion of Sulfateless (Sfl), an enzyme needed for the modification of HS chains within glypicans [53], also rescued the autonomous and nonautonomous effects on tissue growth caused by *PTEN* depletion (Fig 6E). Targeted expression of dsRNA forms of *sfl* in otherwise wild-type wings caused a strong tissue-autonomous size reduction (S2 Fig, blue bars), but little effect was observed in adjacent cell territories (S2 Fig, white bars). Interestingly, depletion of Dally was not able to rescue the nonautonomous reduction in cell size caused by activation of the PI3K/PTEN and TSC/TOR pathways (Fig 6A–6C), reinforcing the proposal that cell size is nonautonomously regulated by the PI3K/PTEN and TSC/TOR pathways in a Dally-independent manner.

Mutations in the *hippo* pathway, originally identified in *Drosophila* by their capacity to induce cell proliferation and organ growth, induce tumors in mouse models and occur in a broad range of human carcinomas, including lung, colorectal, ovarian, and liver cancers (reviewed in [54]). Interestingly, the expression levels of the two existing *Drosophila* glypicans, Dally and Dally-like, are up-regulated upon deregulation of the *hippo/Yorkie* pathway (Fig 5B and [55]). Moreover, simultaneous mutations of both *Dally* and *Dally-like* have been shown to rescue the overgrowth caused by clones of cells overexpressing Yorkie [55]. We thus revisited the contribution of these two glypicans to the autonomous effects on tissue growth caused by deregulation of the hippo/Yorkie pathway and analyzed the potential nonautonomous effects on tissue size of the adjacent cell populations. Similar to what happened to TOR activation, deregulation of the hippo/Yorkie pathway causes strong overgrowth and larval lethality [56]. We thus induced a mild activation of the pathway by expressing a wild-type form of the transcription factor Yorkie. Overexpression of Yorkie in the A or P compartments of the developing wing gave rise to a tissue-autonomous increase in size and, most interestingly, a nonautonomous size reduction of the adjacent wild-type territories (Fig 6D and S2 Fig). Interestingly, both the autonomous and nonautonomous effects on tissue size were rescued by depletion of Dally (Fig 6D). In contrast, depletion of Dally-like, using two different dsRNA forms able to reduce its protein levels, did not have this effect (S2 Fig).

Taken together, the above results indicate that Dally, and not Dally-like, contributes to the tissue-autonomous and nonautonomous effects on tissue growth caused by targeted deregulation of the PI3K/PTEN, TSC/TOR, and hippo/Yorkie pathways. These pathways exert their tissue-autonomous action through Dally, most probably by modulating the spreading of the Dpp morphogen and other secreted growth factors, as Dp110 overexpression was still able to induce some growth in Dpp-depleted tissues (Fig 4O). We hypothesize that the nonautonomous effects are a consequence of withdrawal of Dpp from neighboring cells. In order to reinforce the latter notion, we analyzed the capacity of the Dpp type I receptor Thickveins (Tkv), which has been shown to trap Dpp and limit its spreading [43,57], to phenocopy the nonautonomous effects on tissue growth observed upon targeted deregulation of these pathways. Although the expression levels of Tkv (visualized with both an enhancer and a protein trap) were unaffected by targeted deregulation of the PI3K/PTEN pathway (S3 Fig), targeted expression of a wild-type form of Tkv or a dominant negative form of the receptor able to bind Dpp but unable to transduce the signal (Tkv-DN, [58]) gave rise to a nonautonomous size reduction of the adjacent wild-type territories (S3 Fig, white bars). These results emphasize the role of Dpp in modulating the growth and pattern of different cell populations within a developing organ.

### Dally, a Molecular Bridge between Nutrition and Wing Scaling

**Fig 7:**
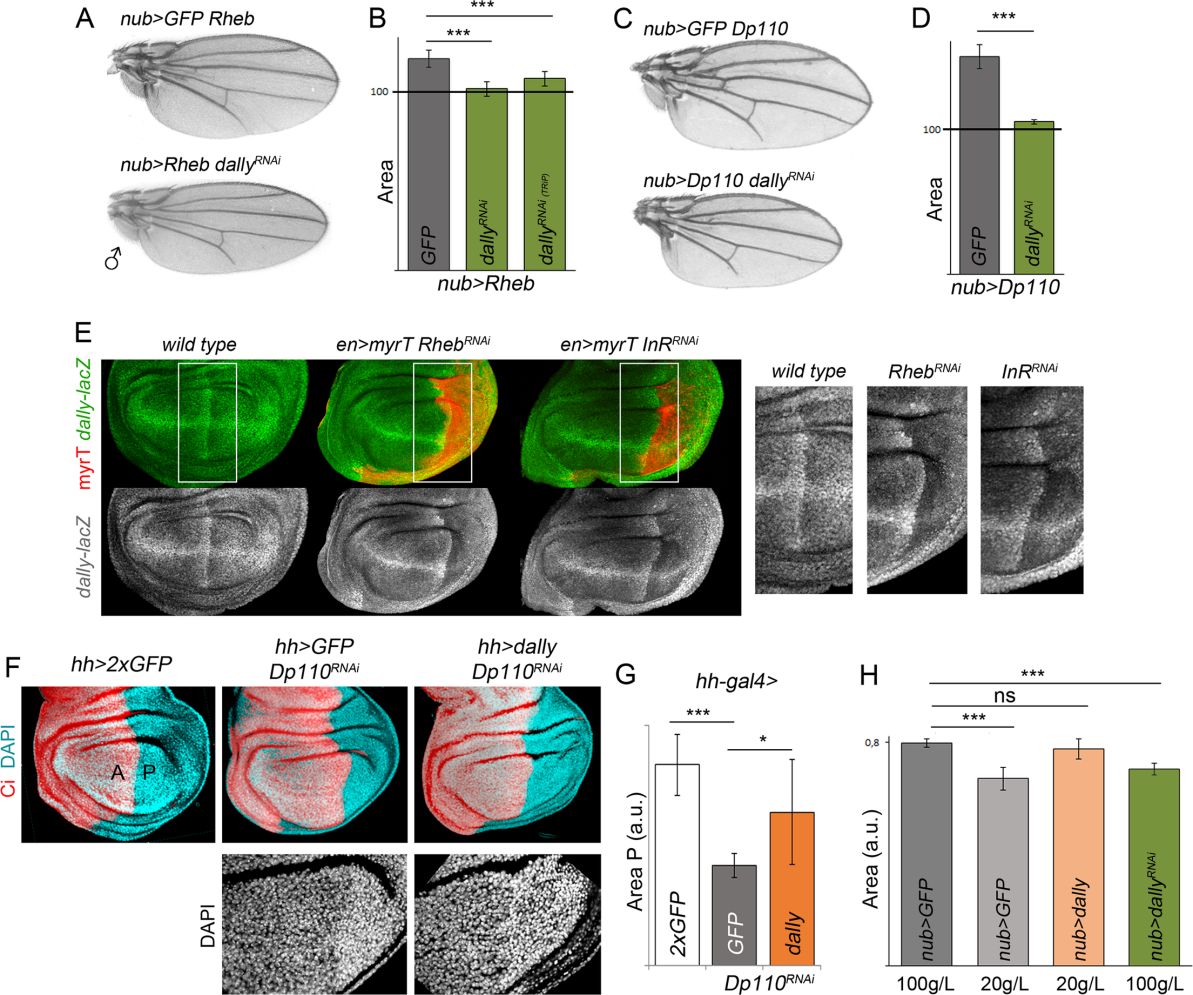
Dally acts as a bridge between nutrient sensing and wing scaling. (A–D) Cuticle preparations (A, C) and histograms plotting tissue size normalized as a percent of the control wings (B, D) of *nub>Rheb* (A, B) or *nub>Dp110* (C, D) adult wings coexpressing either *GFP* or *dally* ^*RNAi*^. Two independent RNA interference (RNAi) lines were used in B. Error bars show the standard deviation. Number of wings analyzed per genotype ≥ 10. ***p < 0.001. (E) Representative wing discs of the indicated genotypes labeled to visualize *dally-lacZ* (antibody to β-Gal, green or white) and myrT expression (in red) to mark the transgene-expressing domain. Higher magnification pictures of the squared regions are shown on the right side. Note the reduced expression of *dally-lacZ* upon depletion of the TSC/TOR or PTEN/PI3K pathways. (F) Representative wing discs of the indicated genotypes and labeled to visualize Ci (red) and DAPI (in blue). Ci labels the anterior compartment. A, anterior compartment; P, posterior compartment. (G) Histogram plotting the P/A size ratio of wing discs of the indicated genotypes. Error bars show the standard deviation. Number of wing discs analyzed per genotype ≥ 10. ***p < 0.001. P/A ratios: hh>2XGFP = 0.55 ± 0.02; hh>GFP, Dp110-RNAi = 0.29 ± 0.06; hh>dally, Dp110-RNAi = 0.42 ± 0.07. (H) Histogram plotting absolute size in a.u. of adult wings of the indicated genotypes. Quantification was made in well-fed (100 g/L yeast food) and starved (20 g/L yeast food) animals. Error bars show the standard deviation. Number of wings analyzed per genotype ≥ 10. ***p < 0.001. ns, not significant.

Besides the roles of the PI3K/PTEN and TSC/TOR signaling pathways in inducing tumor growth [2,4], these two pathways play a conserved role in nutrient sensing and tissue growth during normal development. In the *Drosophila* wing, they modulate the final size of the adult structure according to nutrient availability of the feeding animal (reviewed in [15,16]), and Dpp plays an organ-intrinsic role in the coordination of growth and patterning (reviewed in [17,18]). The identification of the proteoglycan Dally as the rate-limiting factor that contributes to the tissue-autonomous and nonautonomous effects on growth caused by targeted activation of the nutrient-sensing PI3K/PTEN and TSC/TOR pathways suggests that Dally acts as a molecular bridge between the organ-intrinsic and organ-extrinsic mechanisms that regulate organ size. Consistent with this proposal, activation of the PI3K/PTEN or TSC/TOR pathways, which mimics conditions of high nutrient availability, led to an increase in Dally expression levels (Fig 5B) and larger wings (Fig 7A–7D), and depletion of Dally rescued the resulting tissue overgrowth (Fig 7A–7D, see also Fig 6A–6C). These data indicate that Dally contributes to the tissue overgrowth caused by activation of these pathways. We next addressed whether reduced wing size caused by inactivation of the PI3K/PTEN or TSC/TOR pathways, which mimics conditions of low nutrient availability, relies, at least in part, on reduced expression levels of Dally. Interestingly, Dally expression levels were reduced upon targeted depletion of the PI3K/PTEN or TSC/TOR signaling pathways (by expressing dsRNA forms of the insulin-like receptor [InR] and Rheb, respectively; Fig 7E and S4 Fig), and, most interestingly, Dally overexpression was able to partially rescue the tissue size defects caused by depletion of the PI3K/PTEN pathway (Fig 7F and 7G). As expected, the cell size defects were not rescued upon Dally overexpression. These results indicate that the tissue size defects caused by depletion of this pathway rely, at least in part, on the observed reduction in Dally expression levels. Dally overexpression was able only to slightly rescue the tissue size defects of Rheb-depleted wings (S4 Fig), suggesting that other elements are required, together with Dally, to mediate TSC/TOR-dependent tissue growth.

In order to further analyze the role of Dally as a molecular bridge between nutrient-sensing pathways and organ-intrinsic mechanisms, we subjected experimental and control larvae to media containing high (100 g/l yeast) or low (20 g/l yeast) levels of amino acids and analyzed the size of the resulting adult wings upon expression of Dally or a dsRNA form of Dally in the whole wing primordium. Despite the expected reduction in the size of animals reared on food containing 20 g/l yeast, the size of Dally-overexpressing wings (which slightly increases the range of Dpp signaling without affecting the shape of the activity gradient, S4 Fig) was comparable to that of animals receiving food containing 100 g/l yeast (Fig 7H). Similarly, despite the expected increase in animal size reared on food containing 100 g/l yeast, the size of Dally-depleted wings was comparable to that of animals receiving 20 g/l yeast food (Fig 7H). All together, these results reveal that Dally proteoglycan participates in integrating nutrient conditions into wing scaling, most probably through its ability to facilitate the spread of Dpp throughout the tissue.

## Discussion

**Fig 8:**
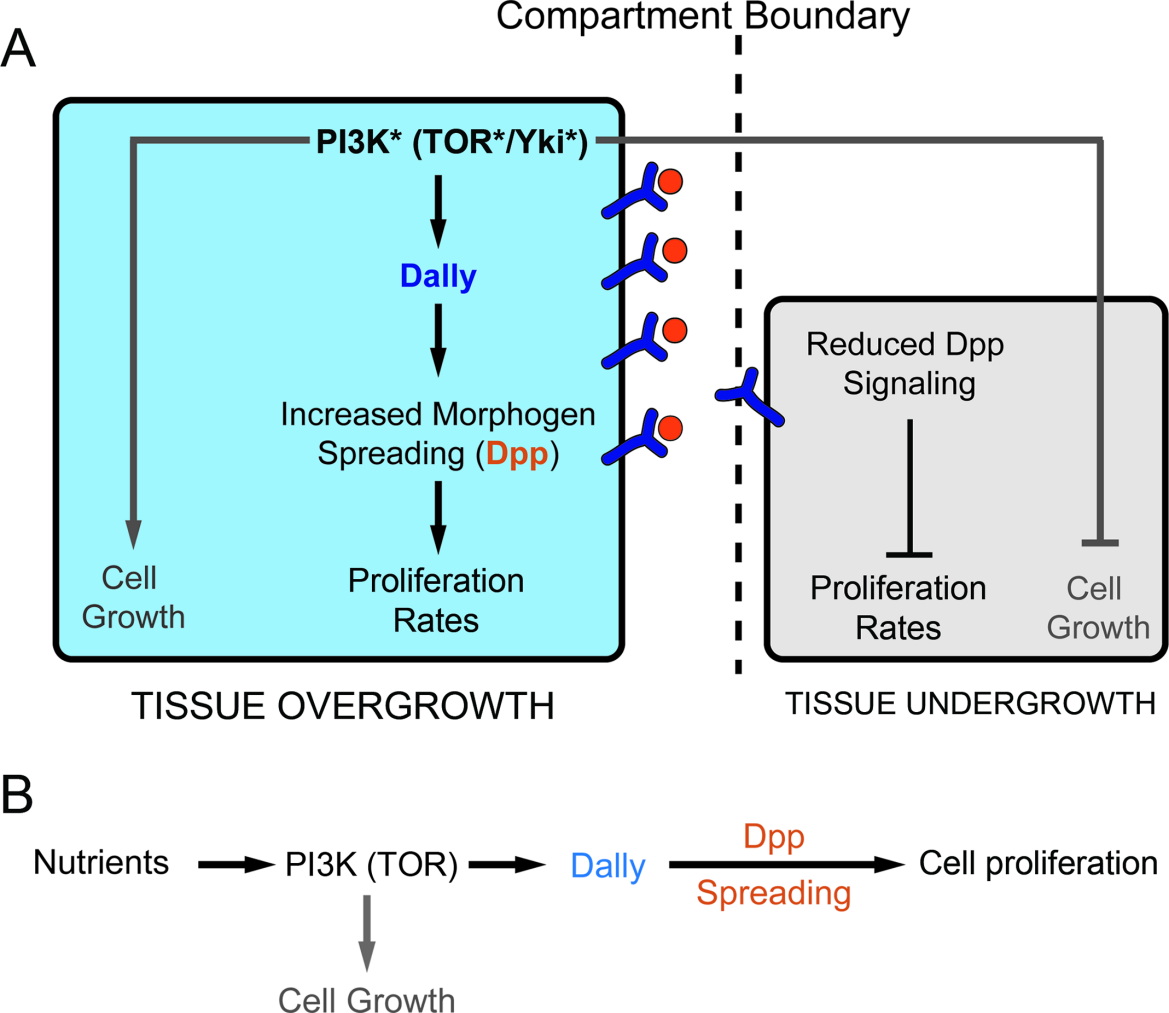
A role of Dally in tissue growth. (A) Activation of the PI3K/PTEN, TSC/TOR, or Yorkie pathways in a defined cell population (in blue) increases cell and/or tissue size in an autonomous manner and induces a nonautonomous reduction in both cell size and number in neighboring cell populations (in grey). Whereas the autonomous and nonautonomous effects on tissue size are mediated by Dally, the effects on cell size are Dally independent. The nonautonomous reduction in tissue size is a consequence of reduced Dpp signaling, most probably reflecting increased number of Dpp molecules bound to the overgrowing (Dally overexpressing) tissue and a consequent reduction in the number of available Dpp molecules to the neighboring cell population. (B) Within the feeding animal, the PI3K/PTEN and TSC/TOR pathways sense nutrient conditions and modulate both cell and tissue size. Whereas the effects on tissue size are mediated by the glypican Dally, which modulates the spreading of the Dpp morphogen throughout the tissue, the effects on cell size are Dally independent.

Here we present evidence that targeted deregulation of the PI3K/PTEN, TSC/TOR, or hippo/Yorkie pathways, known to promote tissue overgrowth by increasing the number and/or size of cells, induces a nonautonomous reduction in tissue size of adjacent cell populations. This nonautonomous effect is a consequence of a reduction in both cell size and proliferation rates (cell number), and it is not a consequence of programmed cell death or the withdrawal of nutrients from neighboring tissues, as reducing the levels of proapoptotic genes or subjecting larvae to different amino-acid diets does not have any impact on the size reduction of neighboring cell populations. We show that the glypican Dally, which plays a major role in regulating the spread of Dpp in *Drosophila* tissues [49–52,59], is up-regulated upon deregulation of these tumor suppressor pathways and that the increase in Dally expression levels contributes to the autonomous effects on tissue size and to the nonautonomous reduction in cell number. Whereas the autonomous effects on tissue size caused by deregulation of these tumor suppressor pathways are most probably due, as least in part (see below), to the capacity of Dally to facilitate Dpp spreading throughout the tissue, we propose that the nonautonomous effects on cell number are a consequence of withdrawal of Dpp from neighboring tissues (Fig 8A). This proposal is based on a number of observations. First, the width of the Dpp activity gradient as well as the total amount of Dpp activity was reduced in adjacent cell populations upon targeted depletion of tumor suppressor pathways. Second, the nonautonomous effects on tissue size were fully rescued by Dally depletion, which has a rather specific role on the spread of Dpp when overexpressed. Third, the nonautonomous effects on tissue size, growth and proliferation rates, and/or Dpp availability and signaling can be phenocopied by overexpression of Dally or the Dpp receptor Tkv. We observed different strengths of the autonomous and nonautonomous effects upon deregulation of these tumor suppressor pathways or overexpression of Dally in either the A or P compartments (Fig 1 and Fig 6). Despite the mild autonomous induction of tissue growth caused by the *ci-gal4* driver in A cells, it caused a relatively strong nonautonomous reduction of the neighboring compartment. On the contrary, the *en-gal4* driver caused a strong autonomous induction of tissue growth in P cells but a relatively weak nonautonomous reduction of the neighboring compartment. The differential autonomous response might simply reflect different strengths of these Gal4 drivers. By contrast, the strongest nonautonomous effects caused by the *ci-gal4* driver (when compared to the *en-gal4* driver) might be because Dpp expression is restricted to the A compartment and increased levels of Dally in Dpp expressing cells are more efficient at titrating out the levels of this growth factor from the neighboring compartment. We noticed that the nonautonomous effects on cell size observed upon deregulation of the PI3K/PTEN, TSC/TOR, or hippo/Yorkie pathways are Dally independent, as overexpression of Dally did not cause a nonautonomous reduction in cell size. Moreover, depletion of Dally did not rescue the nonautonomous reduction in cell size caused by activation of these pathways. These results are consistent with the fact that changes in Dpp signaling do not cause any effect on cell size (S3 Fig, see also [60]) and indicate that Dally and Dpp are regulating cell number but not cell size. Somatic mutations in tumor suppressor genes such as PTEN or TSC are frequently accumulated in early events of tumor development, and these mutations are thought to contribute to the selection of tumorigenic cells. Competition for available growth factors, by modulating the levels of glypicans, such as Dally, might contribute to the outcompetition of wild-type cells and to the selection of malignant mutation-carrying cells in human cancer.

The PI3K/PTEN and TSC/TOR signaling pathways play a role not only in disease but also during normal development. These two pathways modulate the final size of the developing organism according to nutrient availability. Our results also identify, in this context, Dally as a molecular bridge between nutrient sensing and wing scaling in *Drosophila* (Fig 8B). In a condition of high nutrient availability, which leads to the activation of the nutrient-sensing PI3K/PTEN and TSC/TOR pathways, increased levels of Dally facilitate the spread of Dpp throughout the growing tissue and contribute to the generation of larger but well-proportioned and scaled adult structures. Depletion of Dally expression levels rescues the tissue growth caused by high levels of nutrients or activation of the nutrient-sensing pathways and gives rise to smaller and, again, well-proportioned and scaled adult structures. Of remarkable interest is the capacity of Dally to induce tissue overgrowth when overexpressed or to mediate tissue growth upon deregulation of the PI3K/PTEN, TSC/TOR, or hippo/Yorkie pathways. Interestingly, deregulation of these pathways, and the resulting tissue overgrowth, leads to the expansion of the Dpp gradient without affecting the total levels of Dpp signaling. These results imply that Dpp activity levels do not play an instructive role in promoting tissue growth but rather that it is the range of the Dpp gradient that regulates final tissue size. Consistent with this proposal, depletion of Dally levels in one compartment (which might lead to increased levels of available Dpp in the neighboring cell population) does not cause any visible nonautonomous effect in tissue size (S2 Fig). These results are reminiscent of the capacity of Dpp to restrict its own spreading through the repression of Pentagone, a diffusible protein that interacts with Dally and contributes to the expansion of the Dpp gradient [45,46,61]. The graded distribution of Dpp leads, via the interaction with its receptor complex, to the graded activation of Mad/Medea, which in turn represses the transcription of *brinker* (*brk*, reviewed in [42]). This creates a gradient of Brk expression that is reciprocal to the Dpp gradient. Brk is a transcriptional repressor that acts negatively to establish, in a dose-dependent manner, the expression domain of Dpp target genes like *spalt* [62–64]. Thus, Dpp regulates the expression of target genes by repressing *brinker*. Remarkably, the reduced size of the wing primordium observed in hypomorphic alleles of *dpp* is restored when combined with *brk* mutants [65]. This experimental evidence indicates that Dpp controls wing growth entirely via repression of *brk*. The Dally-mediated increase in the width of the Dpp gradient observed upon deregulation of the PI3K/PTEN, TSC/TOR, or hippo/Yorkie pathways might contribute to restrict the expression domain of *brk* to the lateral sides of the wing primordium. Similarly, the nonautonomous decrease in the width of the Dpp gradient might cause an expansion of the *brk* domain, which is known to repress growth. Interestingly, Dally-mediated spreading of other secreted growth factors might also contribute to the autonomous effects on tissue growth caused by deregulation of the PI3K/PTEN, TSC/TOR, or hippo/Yorkie pathways. This is revealed by the fact that Dally depletion rescues both the autonomous and the nonautonomous effects (Fig 6), whereas deregulation of these pathways are still able to induce some growth upon knocking down Dpp (Fig 4O).

Compartments have been proposed to be units of growth control [66]. In other words, the size of each compartment is controlled independently. Our results on the lack of nonautonomous effects on tissue growth upon depletion of Dally or Sfl, the enzyme needed for the modification of HS chains within glypicans, indicate that this is the case. Targeted depletion of glypican expression or activity in the developing compartments gave rise to an autonomous reduction in tissue size without affecting the neighboring compartment (S2 Fig). However, independent lines of evidence support the view that adjacent compartments buffer local variations in tissue growth caused by different means, including a nonautonomous reduction in tissue size upon depletion of the protein biosynthetic machinery [37] or reduced epidermal growth factor receptor (EGFR) activity [67]. Our results on the capacity of overgrowing compartments to withdraw Dpp from neighboring tissues upon targeted deregulation of the PI3K/PTEN, TSC/TOR, or hippo/Yorkie pathways and to cause a nonautonomous reduction in growth and proliferation rates reinforce the view that compartments are susceptible to modulate their growth rates upon different types of stress, including depletion of tumor suppressor genes. Interestingly, the halteres and wings of *Drosophila* are homologous thoracic appendages, and the activity of the *Ultrabithorax* (*Ubx*) *Hox* gene in the haltere discs contributes to defining its reduced size. Remarkably, it does so by reducing the expression levels of *Dally* [68–71], thus reinforcing the role of Dally in modulating tissue growth in epithelial organs.

## Materials and Methods

### *Drosophila* Strains

The *Drosophila* strains used were *UAS–PTEN* ^*RNAi*^ (ID 101475, VDRC); *UAS-Rheb* ^*RNAi*^ (HMS00923, TRiP); *UAS-dally* ^*RNAi*^ (ID 14136, VDRC and HMS00905, TRiP)*; UAS-dlp* ^*RNAi*^ (ID 100268, VDRC; HMS00875, TRiP and HMS00903, TRiP); *UAS-sulfateless* ^*RNAi*^ (ID 5070, VDRC and HMS00543, TRiP)*; UAS-dpp* ^*RNAi*^ (JF01371, TRiP)*; UAS-InR* ^*RNAi*^ (ID 992, VDRC); *UAS-Dp110* ^*RNAi*^ (ID 38985, VDRC); *dally-YFP* (115511, DGRC); *tkv-YFP* (115298, DGRC); *tkv-lacZ* (*P(lacZ)tkv* ^*k16713*^, Bloomington), *UAS-dally* and *dally-lacZ* (*P(lacZ)dally* ^*06464*^, [50], kind gifts from E. Sánchez-Herrero); *UAS-dp53* ^*ct*^ (a dominant negative version of Dp53, [72]); *Df(3L)H99* (a deficiency that covers the pro-apoptotic genes *hid*, *grim*, and *reaper*, [35]); *sal* ^*PE*^ *-Gal4* [47]; *dpp-lacZ* [73]. *en-gal4*, *ci-gal4*, *nub-gal4*, *UAS-dlp*, *UAS-cyclin-E* and *UAS-string* (kind gifts from B. Edgar), *UAS-tkv* and *UAS-tkv* ^*DN*^ (a dominant negative version of tkv that lacked the cytoplasmic kinase domain necessary for signal transduction [58]), *UAS–yki* (a kind gift from S. Cohen), *UAS–Dp110/PI3K* and *UAS-Rheb* are described in Flybase.

### Immunohistochemistry

The antibodies used were rabbit anti-Spalt (a kind gift from R. Barrio, [47]), rabbit anti-pMAD (a kind gift from G. Morata), rabbit anti–phosphorylated histone 3 (PH3) (Cell Signaling), rabbit anti-βgal (Cappel), rat anti-Ci (2A1, DSHB), and mouse anti-Dally-like (13G8, DSHB). Secondary antibodies were obtained from Molecular Probes. TUNEL analysis was performed as described in [74].

### Quantification of Tissue and Cell Size in Adult Wings

The sizes of the wing and of the A and P compartments were measured using Image J Software (NIH, United States). Cell density was measured as the number of hairs (each wing cell differentiates a hair) per defined area. Two conserved regions between veins L4 and L5 (P compartment) and veins L2 and L3 (A compartment) were used to measure cell densities. The final area and cell density values were normalized as a percent of the control values of GFP expressing samples under the corresponding Gal4-drivers. At least ten adult wings per genotype were scored. Only adult males were scored. The average values and the corresponding standard deviation were calculated, and a *t*-test analysis was carried out. The average values, the corresponding standard deviations, and the corresponding *p*-values are included in S1 Data.

### Quantification of Tissue Growth in Developing Wing Discs

Hatched first instar larvae were sorted from egg laying plates (egg laying period of 4 h at 25°C), transferred to fresh tubes, allowed to growth at 25°C, and dissected at three time points of development (72 h, 96 h and 140 h). The size of the A and P compartments in the wing primordia was measured using Fiji Software (NIH, US). At least ten wing discs per genotype and per time point were scored. The corresponding standard deviation was calculated, and a *t*-test analysis was carried out. The average values, the corresponding standard deviations, and the corresponding *p*-values are included in S1 Data.

### Proliferation and Growth Rate Measurements by Clonal Analysis

Early third instar (72 h AEL) larvae of the following genotypes: (1) *hs-FLP*, *ubi-nls-RFP*, *FRT19A/FRT19A*; *ci-gal4*, *UAS-GFP/+*, (2) *hs-FLP*, *ubi-nls-RFP*, *FRT19A/FRT19A*; *ci-gal4*, *UAS-GFP/UAS-Dp110*, and (3) *hs-FLP*, *ubi-nls-RFP*, *FRT19A/FRT19A*; *ci-gal4*, *UAS-GFP/UAS-Dally*, were heat-shocked at 38°C for 1 h and then transferred to 25°C. Wing discs were dissected 72 h after clone induction. The size of clones, visualized by the absence of RFP expression, was quantified from confocal images with ImageJ software (NIH, US). Wing discs were also labelled with DAPI to quantify cell number. At least 30 clones were quantified. Average values and the corresponding standard deviations were calculated, and a *t*-test analysis was carried out. Clones in the three different genotypes were induced and dissected in parallel, and the same control wing discs (genotype nr 1) were used in Figs 2 and 5. The average values, the corresponding standard deviations, and the corresponding *p*-values are included in S1 Data.

### Flow Cytometry Analysis

Wing imaginal discs of each genotype were dissected in cold PBS, dissociated with trypsin-EDTA at 32°C for 45 min, and fixed with 4% formaldehyde for 20 min. Cells were centrifuged at 2,000 rpm for 2 min, resuspended in 1 ml of 70% ethanol, and incubated for 1 h at room temperature (RT). After centrifugation, the pellet was resuspended in PBS with DAPI 1 mg/ml and RNase 100 mg/ml and incubated for 1 h at RT. DAPI fluorescence was determined by flow cytometry using a FacsAria I SORP sorter (Beckton Dickinson, San Jose, California). Excitation with the blue line of the laser (488 nm) permits the acquisition of scatter parameters. Blue laser (488 nm) was used for GFP excitation, and a UV laser (350 nm) for DAPI excitation. Doublets were discriminated using an integral/peak dot plot of DAPI fluorescence. Cell cycle histograms were obtained on each sample according to the GFP fluorescence, and cell cycle analysis was done on DAPI fluorescence collected at 440 nm. DNA analysis on single fluorescence histograms was done using Summit software (Dako Colorado).

### pMAD Expression Profile

In order to obtain the pMAD expression profile at the same developmental stage in all genotypes analyzed, larvae were fed with food complemented with bromophenol blue (0,2 g in 100 ml of fly food), as described in [75], and wandering larvae with empty (white) gut, corresponding to 1–6 h before the entry into pupal stage, were used to dissect the wing discs. As previously described in [45], all wing discs from a dataset (GFP control and experimental discs) were fixed and stained together to avoid variability between discs and mounted on the same slide to reduce potential variation in thickness between the slide and the coverslip across different samples. All discs from a dataset were imaged under identical settings using a Leica SP2 confocal microscope (0.36 μm of step size). Confocal conditions were adjusted to avoid saturated pixels with maximal intensity. After image acquisition, four consecutive slices (above and below the brightest slice from each stack) were manually selected by visual inspection, and a mean projection of these nine slices was subsequently performed. Using a reduced number of slices and performing the mean projection allowed us to reduce the noise as well as to avoid the signal from the peripodial membrane. Indeed, we made sure that these nine slices contained signal from the columnar cells of the pouch only. All discs were rotated to have anterior to the left and dorsal upwards orientation. The remaining analyses were applied solely to the wing pouch. We extracted the profiles along the anterior–posterior (AP) axis parallel to the dorsal-ventral (DV) boundary in four different offsets (two in the dorsal compartment and two in the ventral one). The positioning of the AP boundary was scored by the GFP expression driven by the *gal4* line. Data are included in S1 Data. To quantify total pMAD signaling of a particular region of interest (A or P compartments or the whole wing pouch), signal intensities per pixel were first collected from raw images taken with the same laser confocal conditions using the histogram function of Fiji Software (NIH, US). Confocal conditions were adjusted to avoid saturated pixels with maximal intensity. Total pMAD signaling per region of interest was quantified as the sum of all pixel intensities. All wing discs from a dataset (GFP control and experimental discs) were fixed and stained together. All of the image processing and data extraction were performed using Fiji Software (NIH, USA). At least five discs per genotype were analyzed. The average values, the corresponding standard deviations, and the corresponding *p*-values are included in S1 Data.

### Fly Food with Varying Concentrations of Yeast

We adapted the food recipe described in [76]. The following recipes were used to vary the concentration of yeast: (A) 100 g/L yeast food: 1 liter of standard fly medium contains 100 g of fresh yeast, 100 g of sucrose, 27 g bacto agar, 3 mL of propionic acid, and 30 mL of nipagin; and (B) 20 g/L yeast food (starvation): it was generated by reducing the amount of fresh yeast without altering the other ingredients.

Hatched first instar larvae were sorted from egg laying plates (egg laying period of 8 h at 25°C), transferred to tubes with the different yeast concentration foods and allowed to grow at 25°C to adulthood. Control animals were analyzed in parallel in each experimental condition.

### Statistical Analyses

In all quantifications, the Student’s *t*-test (two-tailed) was used to test for significance. Significance is indicated in the figures using the following symbols: *p < 0.05, **p < 0.01, ***p < 0.001. Error bars represent either the standard deviation (in the case of tissue size and cell density measurements) or the standard error to the mean (in the case of quantification of mitotic figures, apoptotic cells, and pMAD signal intensity).

## Supporting Information

S1 Data Quantitative measurements with their corresponding standard deviations of tissue size and/or cell density values in adult wings (Figs 1–3, Figs 5–7, and S1–S4 Figs) or wing imaginal discs (Fig 4 and Fig 7), clonal area and number of cells per clone (Fig 2 and Fig 5), number of apoptotic cells (Fig 3 and S1 Fig), pMAD intensity (Fig 4, Fig 5, and S2 Fig), and mitotic figures (S1 Fig) of individuals expressing the indicated transgenes under the control of the corresponding Gal4 drivers. A *t*-test was carried out to calculate the *p*-value as a measurement of the statistical significance. Data are ordered with respect to the corresponding main or supplementary figures. Further tabs present the underlying numerical data used to generate the pMAD profiles shown in Figs 4E, 4F, 4H, 4I, 4L, and 5J and S2A, S2J, S2K and S4C Figs.
(XLSX)

S1 Fig Nonautonomous effects on tissue size upon targeted activation of PI3K/PTEN and TSC/TOR pathways. (A) Cuticle preparations of male adult wings expressing *GFP* or *PTEN* ^*RNAi*^ under the control of the *hh-gal4* driver. The blue line marks the boundary between the anterior (A) and posterior (P) compartments. (B–D) Histograms plotting tissue size of the whole wing (B) and tissue size and cell density of A (white bars) and P (blue bars) compartments (C, D) of adult wings expressing the indicated transgenes in the *hh* domain normalized as a percent of the control wings. Note a consistent reduction in tissue size of the adjacent cell populations (white bars). Error bars indicate the standard deviation. Number of wings analyzed per genotype ≥ 10. ***p < 0.001. (E, F) *ci>GFP*, *CycE*, *string* wing imaginal discs labelled with TUNEL to visualize apoptotic cells (in red, E) or with an antibody against phosphorylated histone 3 (PH3, in magenta or white) to visualize mitotic cells (F). The *ci* domain is labelled with GFP (in green). (G, H) DNA-content profile of FACS-sorted GFP-expressing and nonexpressing cells dissociated from *ci>GFP* and *ci>GFP*, *Dp110* wing imaginal discs. Percentage of cells in G1, G2, and S is indicated. (I) Histogram plotting the quantification of mitotic figures per area in the A (grey bars) and P (blue bars) compartments of the indicated genotypes. Error bars indicate the standard deviation. Number of wing discs analyzed per genotype ≥ 10. ***p < 0.001; **p < 0.01; *p < 0.05. (J) Histogram plotting the quantification of the absolute number of TUNEL-positive cells in the P (light green bars) and A (grey bars) compartments of the indicated genotypes. Error bars indicate the standard deviation. Number of wing discs analyzed per genotype ≥ 10. ***p < 0.001.
(TIF)

S2 Fig Glypicans and tissue growth. (A) Average pMAD profile of wing discs expressing *GFP* (red line) or *GFP* and the corresponding transgenes (blue line) under the control of the *en-gal4* driver. Profiles were taken along the AP axis and plotted in absolute positions. The standard error to the mean is shown in the corresponding color for each genotype. The AP boundary of both experiments was aligned to allow comparison of the profile in each compartment. Number of wing discs analyzed per genotype ≥ 5. The domains of transgene expression are marked with a black asterisk. Arrows mark the limits of the Dpp activity gradients. (A’) Histograms plotting the total intensity of pMAD signal in a.u. of the posterior (blue bars) and anterior (white bars) compartments of *en>2xGFP* and *en>GFP*, *PTEN* ^*RNAi*^ wing discs. Error bars indicate the standard error to the mean. Number of wing discs analyzed per genotype ≥ 5. *p < 0.05 (B) Wing imaginal discs of the indicated genotypes labelled to visualize Dally expression. Expression of Dally was analyzed in flies carrying *a Dally-YFP* reporter. Red brackets indicate the domain of transgene (*myrT* or *PTEN* ^*RNAi*^) expression. High magnification of the squared region is shown in the right panel. (C–H) On the left, cuticle preparations of adult wings of the indicated genotypes. On the right, histograms plotting tissue size (C–H) and cell density (C, D) values of the transgene-expressing compartment (blue bars) and adjacent compartment (white bars), normalized as a percent of the control wings. Error bars show the standard deviation. Number of wings analyzed per genotype ≥ 10. ***p < 0.001; **p < 0.01; *p < 0.05. The blue line marks the boundary between the A and P compartments, and the domains of transgene expression are marked with a blue asterisk. (I) Wing imaginal disc of the indicated genotype labelled to visualize Dally-like (in red or white) and GFP (in green) protein expression. (J) Average pMAD profiles of wing discs expressing *GFP* and Dp110 (blue line) or *GFP*, a dsRNA form against *Dally* and *Dp110* (green line) under the control of the *ci-gal4* driver. Profiles were taken along the AP axis and plotted in absolute positions. The standard error to the mean is shown in the corresponding color for each genotype. The AP boundary of both experiments was aligned to allow comparison of the profile in each compartment. Number of wing discs analyzed per genotype ≥ 5. The domains of transgene expression are marked with a black asterisk. Arrows mark the limits of the Dpp activity gradients. (J’) Histograms plotting the total intensity of pMAD signal in a.u. of the anterior (blue bars) and posterior (white bars) compartments of *ci>GFP*, *Dp110* and *ci>Dp110*, *Dally-RNAi* wing discs. Error bars indicate the standard error to the mean. Number of wing discs analyzed per genotype ≥ 5. (K) Wing imaginal discs (left panels) of *ci>GFP* and *ci>dally* ^*RNAi*^ larvae labelled to visualize pMAD protein (in white) and average pMAD profiles (right panels) of wing discs expressing *GFP* (red line) or *dally* ^*RNAi*^ (green line) in the *ci* domain. Profiles were taken along the AP axis and plotted in absolute positions. The standard error to the mean is shown in the corresponding color for each genotype. The AP boundary of both experiments was aligned to allow comparison of the profile in each compartment. Number of wing discs analyzed per genotype ≥ 7. The domains of transgene expression are marked with a black asterisk. Arrows mark the limits of the Dpp activity gradients.
(TIF)

S3 Fig Thickveins and tissue growth. (A, B) Wing imaginal discs of the indicated genotypes labelled to visualize *thickveins* expression. Expression of *thickveins* was analyzed in flies carrying *tkv-lacZ* (A) or *tkv-YFP* (B) reporters. Red brackets indicate the domain of transgene (*myrT* or *PTEN* ^*RNAi*^) expression. (C, D) On the left, cuticle preparations of adult wings of the indicated genotypes. On the right, histograms plotting tissue size (C,D) and cell density (D) values of the transgene-expressing compartment (blue bars) and of the adjacent compartment (white bars), normalized as a percent of the control wings. Error bars show the standard deviation. Number of wings analyzed per genotype ≥ 10. ***p < 0.001; **p < 0.01; *p < 0.05. The blue line marks the boundary between the anterior (A) and posterior (P) compartments, and the domains of transgene expression are marked with a blue asterisk.
(TIF)

S4 Fig Regulation of Dally by the PI3K and TOR pathways. (A) Wing imaginal discs of the indicated genotypes labelled to visualize Dally expression (in red or white) and DAPI (in blue to visualize nuclei). The transgene-expressing domain is labelled with GFP (in green). Expression of Dally was analyzed in flies carrying *a Dally-lacZ* reporter. (B) Histogram plotting tissue size and cell density values normalized as a percent of the control (*nub>GFP*) of *nub>Rheb* ^*RNAi*^ adult wings coexpressing either *GFP* or *Dally*. Error bars show the standard deviation. Number of wings analyzed per genotype ≥ 10. *p < 0.05. (C) Wing imaginal discs (left panels) of *nub>GFP* and *nub>dally* larvae labelled to visualize pMAD protein (in red) and average pMAD profiles (right panels) of wing discs expressing *GFP* (red line) or *Dally* (blue line) in the *nubbin* domain. Profiles were taken along the AP axis and plotted in absolute positions. The standard error to the mean is shown in the corresponding color for each genotype. The AP boundary of both experiments was aligned to allow comparison of the profile in each compartment. Number of wing discs analyzed per genotype ≥ 7. Arrows mark the limits of the Dpp activity gradients.
(TIF)

## Abbreviations

βGal: β-galactosidase
A: anterior
AEL: after egg laying
AP: anterior–posterior
a.u.: arbitrary units
*brk*: *brinker*
*cycE*: *cyclin E*
Dpp: Decapentaplegic
DSHB: Developmental Studies Hybridoma Bank
dsRNA: double-stranded RNA
DV: dorsal-ventral
EGFR: epidermal growth factor receptor
FACS: fluorescence-activated cell sorter
GFP: green fluorescent protein
GPI: glycosylphosphatidylinositol
GTP: guanosine-5'-triphosphate
HS: heparan sulfate
HSPG: heparan sulfate proteoglycan
InR: insulin-like receptor
MAD: Mothers against Dpp
P: posterior
PH3: phosphorylated histone H3
PI3K: phosphatidylinositol 3-kinase
PTEN: phosphatase and tensin homolog
RFP: red fluorescent protein
RNAi: RNA interference
RT: room temperature
Sfl: Sulfateless
*stg*: *string*
TGF-β: transforming growth factor β
Tkv: Thickveins
TOR: target of rapamycin
TSC: tuberous sclerosis complex
TUNEL: terminal deoxynucleotidyl transferase dUTP nick end labeling
UAS: upstream activation sequence
*Ubx*: *Ultrabithorax*
VDRC: Vienna Drosophila RNAi Center
