# Supplementary figures and images for: Dally Proteoglycan Mediates the Autonomous and Nonautonomous Effects on Tissue Growth Caused by Activation of the PI3K and TOR Pathways
Source: PLoS Biol. 2015 Aug 27;13(8):e1002239. doi: 10.1371/journal.pbio.1002239 (PMC4551486; doi:10.1371/journal.pbio.1002239)

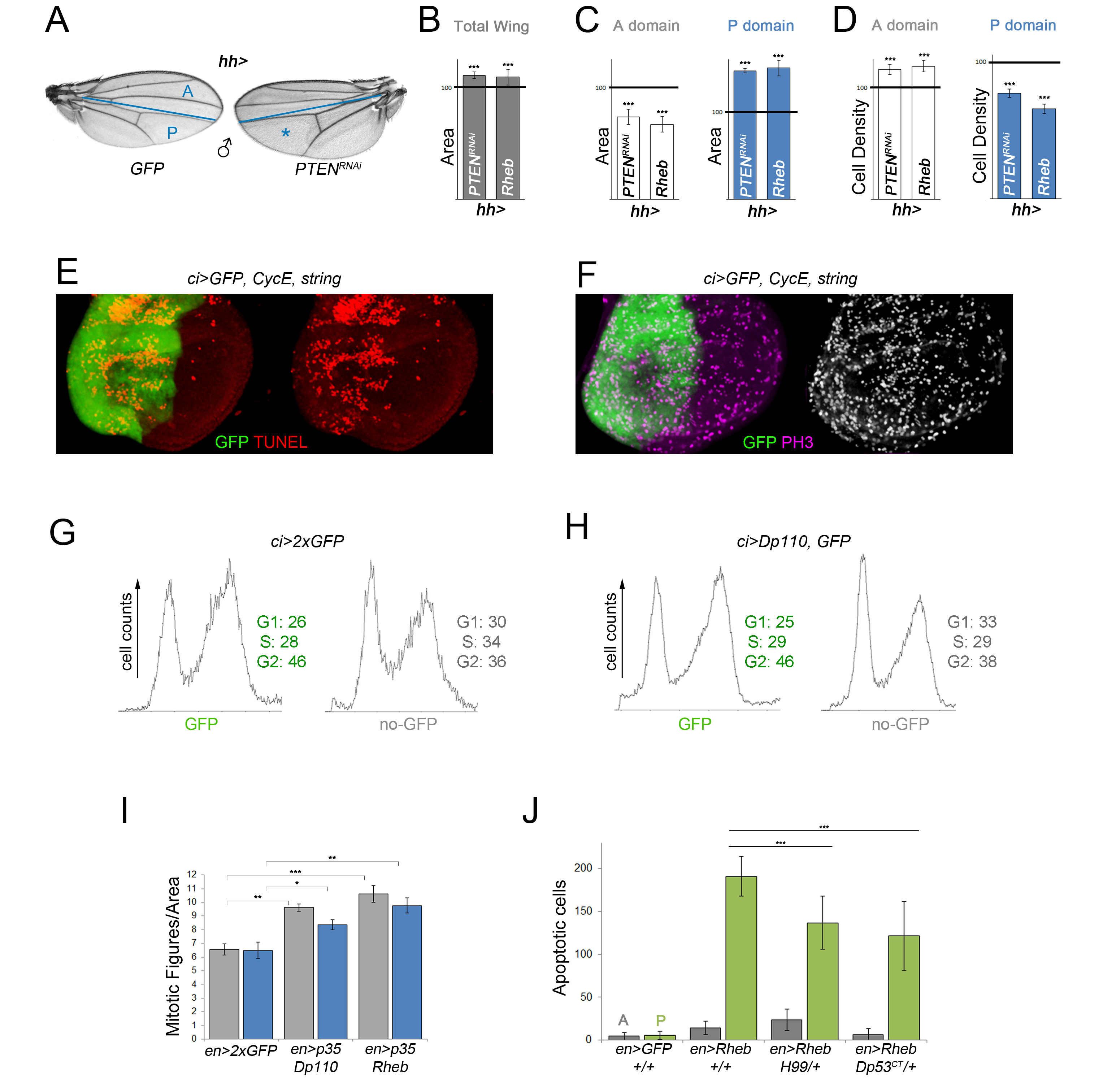

Supplement: S1 Fig — (A) Cuticle preparations of male adult wings expressing GFP or PTEN RNAi under the control of the hh-gal4 driver. The blue line marks the boundary between the anterior (A) and posterior (P) compartments. (B–D) Histograms plotting tissue size of the whole wing (B) and tissue size and cell density of A (white bars) and P (blue bars) compartments (C, D) of adult wings expressing the indicated transgenes in the hh domain normalized as a percent of the control wings. Note a consistent reduction in tissue size of the adjacent cell populations (white bars). Error bars indicate the standard deviation. Number of wings analyzed per genotype ≥ 10. ***p < 0.001. (E, F) ci>GFP, CycE, string wing imaginal discs labelled with TUNEL to visualize apoptotic cells (in red, E) or with an antibody against phosphorylated histone 3 (PH3, in magenta or white) to visualize mitotic cells (F). The ci domain is labelled with GFP (in green). (G, H) DNA-content profile of FACS-sorted GFP-expressing and nonexpressing cells dissociated from ci>GFP and ci>GFP, Dp110 wing imaginal discs. Percentage of cells in G1, G2, and S is indicated. (I) Histogram plotting the quantification of mitotic figures per area in the A (grey bars) and P (blue bars) compartments of the indicated genotypes. Error bars indicate the standard deviation. Number of wing discs analyzed per genotype ≥ 10. ***p < 0.001; **p < 0.01; *p < 0.05. (J) Histogram plotting the quantification of the absolute number of TUNEL-positive cells in the P (light green bars) and A (grey bars) compartments of the indicated genotypes. Error bars indicate the standard deviation. Number of wing discs analyzed per genotype ≥ 10. ***p < 0.001. (TIF) [file pbio.1002239.s002.tif]

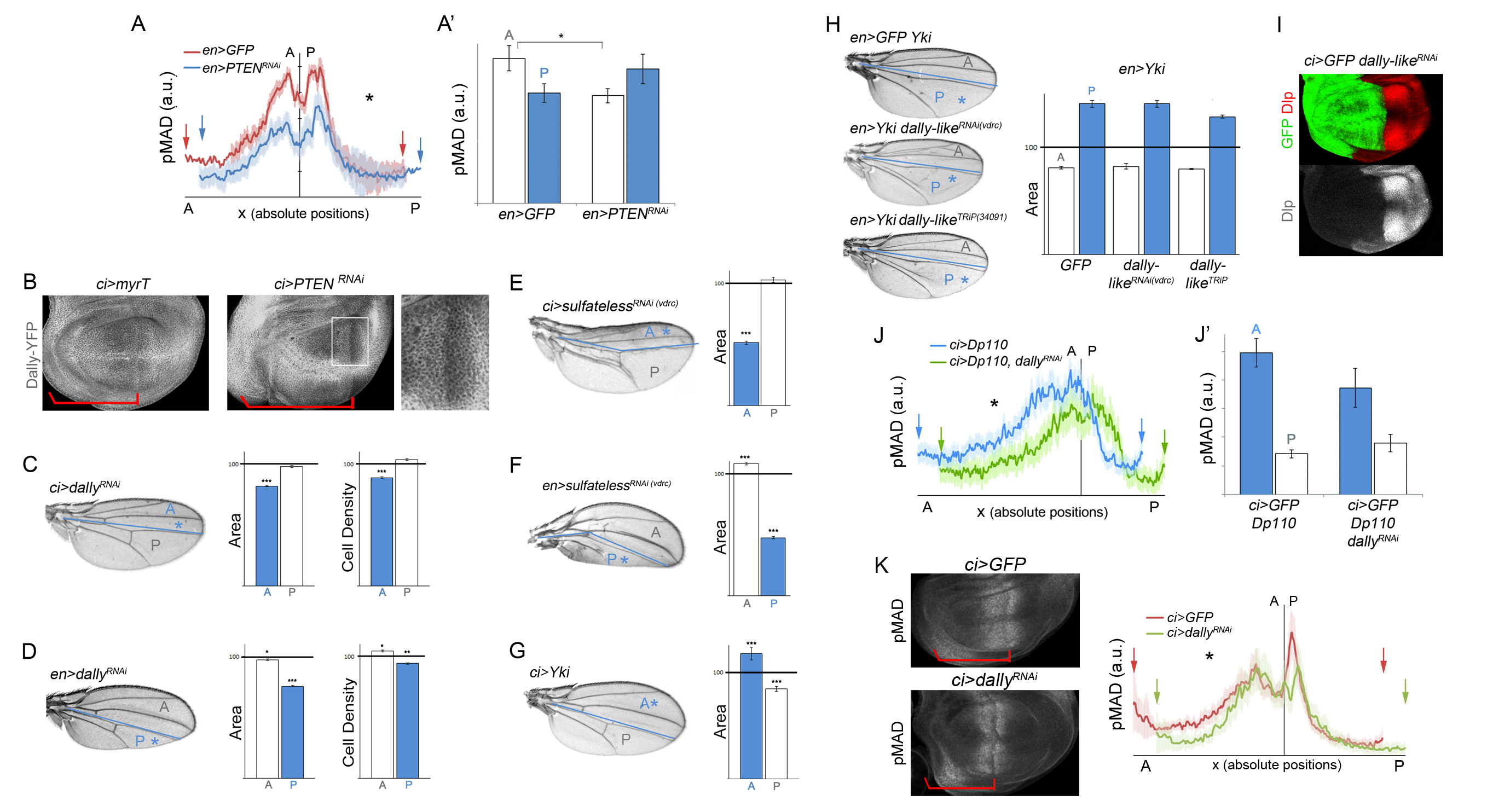

Supplement: S2 Fig — (A) Average pMAD profile of wing discs expressing GFP (red line) or GFP and the corresponding transgenes (blue line) under the control of the en-gal4 driver. Profiles were taken along the AP axis and plotted in absolute positions. The standard error to the mean is shown in the corresponding color for each genotype. The AP boundary of both experiments was aligned to allow comparison of the profile in each compartment. Number of wing discs analyzed per genotype ≥ 5. The domains of transgene expression are marked with a black asterisk. Arrows mark the limits of the Dpp activity gradients. (A’) Histograms plotting the total intensity of pMAD signal in a.u. of the posterior (blue bars) and anterior (white bars) compartments of en>2xGFP and en>GFP, PTEN RNAi wing discs. Error bars indicate the standard error to the mean. Number of wing discs analyzed per genotype ≥ 5. *p < 0.05 (B) Wing imaginal discs of the indicated genotypes labelled to visualize Dally expression. Expression of Dally was analyzed in flies carrying a Dally-YFP reporter. Red brackets indicate the domain of transgene (myrT or PTEN RNAi) expression. High magnification of the squared region is shown in the right panel. (C–H) On the left, cuticle preparations of adult wings of the indicated genotypes. On the right, histograms plotting tissue size (C–H) and cell density (C, D) values of the transgene-expressing compartment (blue bars) and adjacent compartment (white bars), normalized as a percent of the control wings. Error bars show the standard deviation. Number of wings analyzed per genotype ≥ 10. ***p < 0.001; **p < 0.01; *p < 0.05. The blue line marks the boundary between the A and P compartments, and the domains of transgene expression are marked with a blue asterisk. (I) Wing imaginal disc of the indicated genotype labelled to visualize Dally-like (in red or white) and GFP (in green) protein expression. (J) Average pMAD profiles of wing discs expressing GFP and Dp110 (blue line) or GFP, a dsRNA form ag [file pbio.1002239.s003.tif]

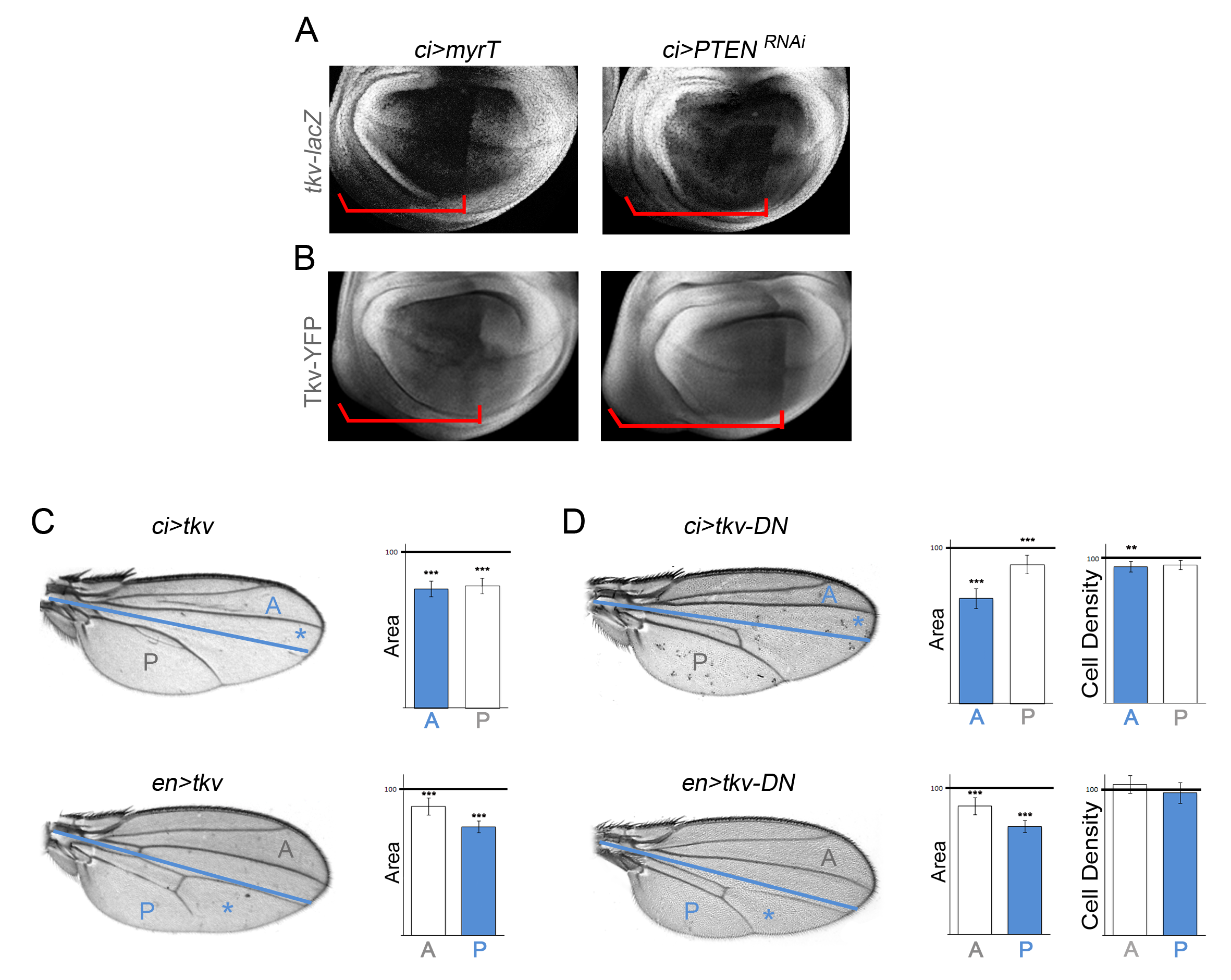

Supplement: S3 Fig — (A, B) Wing imaginal discs of the indicated genotypes labelled to visualize thickveins expression. Expression of thickveins was analyzed in flies carrying tkv-lacZ (A) or tkv-YFP (B) reporters. Red brackets indicate the domain of transgene (myrT or PTEN RNAi) expression. (C, D) On the left, cuticle preparations of adult wings of the indicated genotypes. On the right, histograms plotting tissue size (C,D) and cell density (D) values of the transgene-expressing compartment (blue bars) and of the adjacent compartment (white bars), normalized as a percent of the control wings. Error bars show the standard deviation. Number of wings analyzed per genotype ≥ 10. ***p < 0.001; **p < 0.01; *p < 0.05. The blue line marks the boundary between the anterior (A) and posterior (P) compartments, and the domains of transgene expression are marked with a blue asterisk. (TIF) [file pbio.1002239.s004.tif]

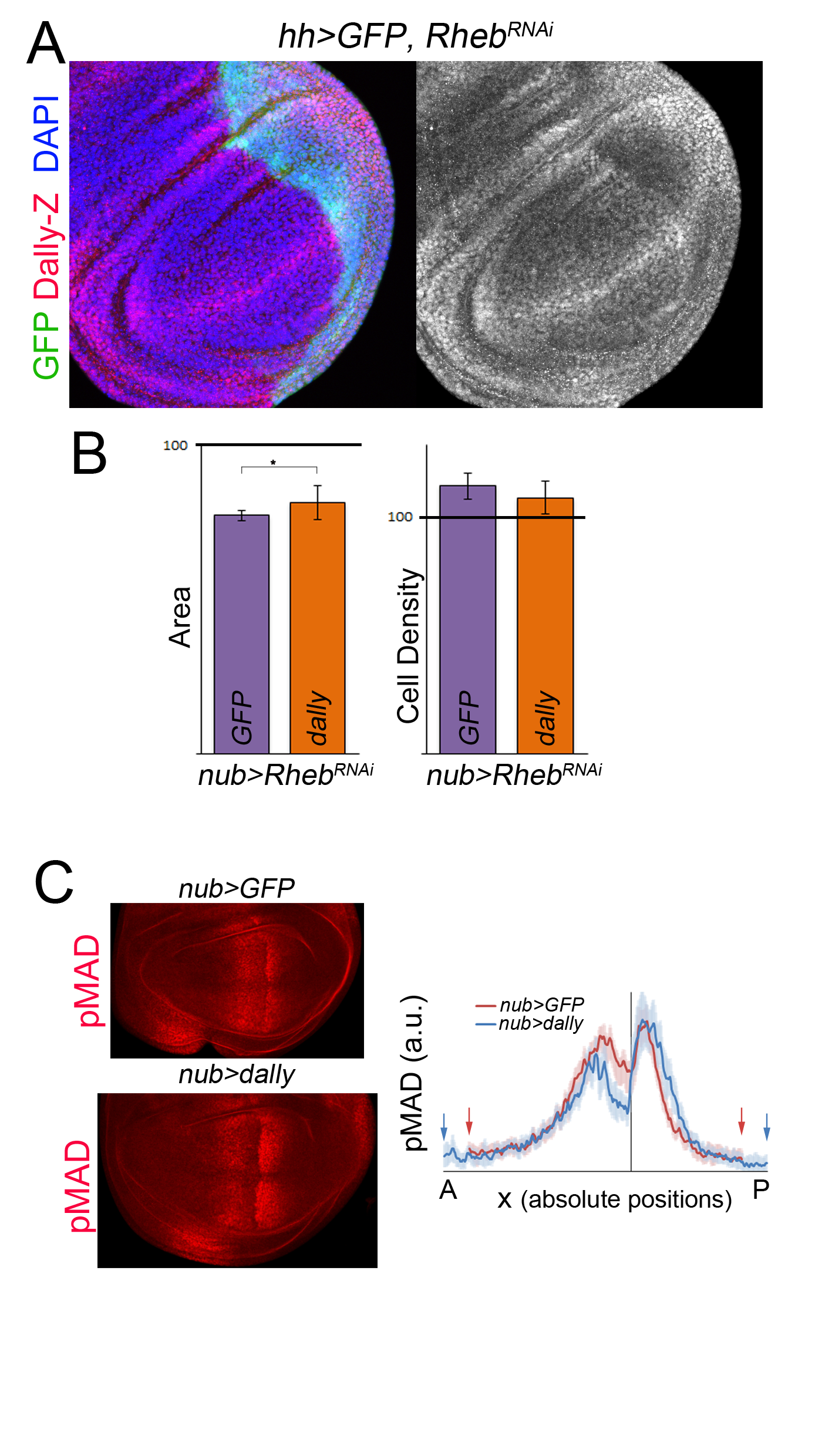

Supplement: S4 Fig — (A) Wing imaginal discs of the indicated genotypes labelled to visualize Dally expression (in red or white) and DAPI (in blue to visualize nuclei). The transgene-expressing domain is labelled with GFP (in green). Expression of Dally was analyzed in flies carrying a Dally-lacZ reporter. (B) Histogram plotting tissue size and cell density values normalized as a percent of the control (nub>GFP) of nub>Rheb RNAi adult wings coexpressing either GFP or Dally. Error bars show the standard deviation. Number of wings analyzed per genotype ≥ 10. *p < 0.05. (C) Wing imaginal discs (left panels) of nub>GFP and nub>dally larvae labelled to visualize pMAD protein (in red) and average pMAD profiles (right panels) of wing discs expressing GFP (red line) or Dally (blue line) in the nubbin domain. Profiles were taken along the AP axis and plotted in absolute positions. The standard error to the mean is shown in the corresponding color for each genotype. The AP boundary of both experiments was aligned to allow comparison of the profile in each compartment. Number of wing discs analyzed per genotype ≥ 7. Arrows mark the limits of the Dpp activity gradients. (TIF) [file pbio.1002239.s005.tif]
